# Beyond Metal‐Air Chemistry: Coupling n‐/p‐Type Organic Redox Chemistry Toward Sustainable Sub‐Zero‐Temperature All‐Polymer Seawater Batteries

**DOI:** 10.1002/advs.202504004

**Published:** 2025-08-04

**Authors:** Yuanzhe Lu, Xiu Liu, Fan Yang, Zishou Zhang, Fei Xin, Linfeng Zhong, Dingshan Yu

**Affiliations:** ^1^ Key Laboratory for Polymeric Composite and Functional Materials of Ministry of Education Key Laboratory of High‐Performance Polymer‐based Composites of Guangdong Province GBRCE for Functional Molecular Engineering School of Chemistry School of Chemical Engineering and Technology Sun Yat‐sen University Guangzhou Guangzhou 510006 China; ^2^ School of Light Industry Science and Engineering Beijing Technology and Business University Beijing China

**Keywords:** all‐polymer batteries, organic electrodes, organic redox chemistry, redox polymers, seawater batteries

## Abstract

Developing high‐efficiency durable rechargeable seawater battery (RSB) that directly harnesses natural seawater for sustainable energy storage is an attractive yet challenging task. Rather than use traditional metal‐air chemistry, for the first time it proposes a coupled n‐type/p‐type redox chemistry to establish a new all‐polymer RSB (APRSB) with high energy efficiency and long cycle lifespan even at subzero temperature. The strategy involves rational design of n‐type redox polyimide (NDIP) and p‐type redox radical polymer (RRP). By using flexible triamine linker to crosslink naphthalenetetracarboxylic dianhydride (NTCDA) molecules to tune aggregate structures, the newly‐produced NDIP attains consistent high storage capacity for multiple cations (Na^+^/K^+^/Mg^2+^/Ca^2+^), enabling seawater‐adaptable low‐potential anodes with fast redox kinetics and large specific capacity along with long lifespan over 10 000 cycles—superior to most previous electrodes in seawater. By exerting inherent advantages of radicals, RRP is explored as seawater‐suitable well‐matched high‐potential cathodes, integrating facile redox property and superior adaptability with SO_4_^2−^ and even the corrosive Cl^−^. Thus, paring NDIP and RRP yields APRSB with high output voltage, large energy efficiency (81%), and 83.2% capacity retention after 800 cycles even at −4 °C—outperforming most previous room‐temperature seawater batteries. Detailed studies reveal facilitated organic redox kinetics in seawater resistive against low temperature.

## Graphical abstract

A coupled n‐type/p‐type redox chemistry instead of conventional metal‐air chemistry has for the first time been proposed to develop a new family of all‐polymer rechargeable seawater battery, displaying high energy efficiency, excellent rate capability and cycling stability even at low temperature.

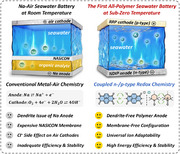

## 1 Introduction

**Figure 1:**
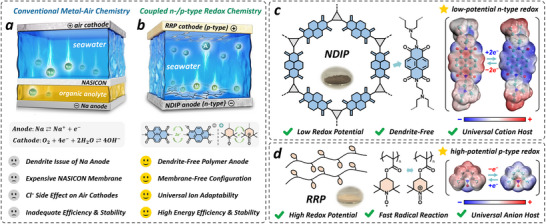
Schematic configurations of a) conventional half‐seawater batteries based on Na‐air chemistry with NASICON membranes and b) our proposed all‐polymer seawater battery coupled n‐/p‐type chemistry. Envisioned structure illustrations, redox mechanism, molecular electrostatic potential maps, and the actual sample photos of c) NDIP and d) RRP.

The ever‐growing energy and environmental crises necessitate the development of eco‐friendly and cost‐effective next‐generation sustainable energy storage technologies aimed at directly utilizing earth‐abundant natural resources.^[^ ^1^, ^2^, ^3^, ^4^ ^]^ In this regard, the newly‐emerging rechargeable seawater batteries (RSBs) that directly employ natural seawater as electrolytes are particularly attractive owing to their distinct advantages: (1) inherent safety and reduced assembly cost enabled by the inexhaustible seawater supply (≈96.5% of the total water on earth); (2) high ionic conductivity of seawater (≈50 mS cm^−1^); (3) expanded application scope to seaside and offshore energy storage.^[^ ^5^, ^6^ ^]^ Early RSBs pioneered by Kim et al.^[^ ^7^, ^8^ ^]^ comprise a Na anode in non‐aqueous electrolyte and an air‐cathode in seawater electrolyte separated by a Na^+^ single‐ion conductive membrane (NASICON), involving the plating/stripping process of Na metal at the anode and the oxygen evolution/reduction reaction at the cathode during charge/discharge, i.e., the so‐called metal‐air chemistry (**Figure** **1**a). Despite great promise, these half‐seawater batteries face several critical challenges: dendritic growth on the Na anode; reliance on expensive NASICON membranes with high ionic resistance; inherently sluggish oxygen kinetics in near‐neutral seawater; and detrimental effects of chloride ions (Cl^−^), which can block active sites on the air catalyst and accelerate its degradation.^[^ ^9^ ^]^ These issues undoubtedly constrain the energy efficiency (typically <60%) and cycle life (<200 cycles) of RSBs. To mitigate these challenges, various strategies have been proposed, such as replacing air‐cathode chemistry with chloride capture/release or intercalation/deintercalation reactions, for increased energy efficiency. Still, the cycling life and rate capability of RSBs are inadequate, limited by the metal anodes. More recently, a lattice‐expanded TiO_2_ has been explored as an appealing substitute for Na anodes to directly store cations in seawater, allowing for NASICON‐free full‐seawater RSBs with prolonged device lifespan;^[^ ^10^ ^]^ however, the overall output voltage and energy efficiency remain suboptimal due to the persistent issue of sluggish air‐cathode kinetics.^[^ ^11^, ^12^, ^13^ ^]^ Despite encouraging progress, the RSB is still at an early stage, and developing new battery paradigm beyond metal‐air chemistry to simultaneously improve both anode and cathode for high‐energy‐efficiency full‐seawater batteries with long‐term durability is highly required but rather challenging. Besides, enabling low‐temperature‐adaptable RSB is of great significance for the marine energy storage in high‐latitude regions, yet the feasibility of RSBs at sub‐zero temperatures is rarely explored to date.

Compared to rigid inorganic electrode materials commonly explored as alternatives to metal‐anode or air‐cathode chemistry in RSBs, the flexible redox‐active polymers (RAPs) offer several notable advantages, including low cost, structural diversity, and easy modification via molecule design.^[^ ^14^, ^15^, ^16^, ^17^, ^18^, ^19^, ^20^ ^]^ Generally, organic redox reactions in RAPs enable rapid electron‐transfer kinetics, which are usually not dependent on the counter‐ion type.^[^ ^21^, ^22^, ^23^, ^24^ ^]^ In this context, it is feasible to design one RAP capable of simultaneously uptaking multiple different cations or anions with fast kinetics and thus adaptable for seawater with complex ionic environments. Considering the redox potential and targeted electrode function, the proper RAP can be readily selected and designed. Specifically speaking, by rational molecular design, low‐potential n‐type RAP can be customized to replace the highly‐reactive metal anode to address the dendrite and safety concerns, while high‐potential p‐type RAP serves as a promising candidate for tackling the cyclability and efficiency issues of air cathodes. Building on this concept, we conclude that the coupled n‐type/p‐type organic redox chemistry is anticipated to be a feasible alternative to metal‐air chemistry for developing a new family of all‐polymer seawater batteries, which offers a pathway toward low‐cost, safe, and sustainable energy storage solutions, yet such systems have not been realized to date. Nonetheless, designing both polymer anode and cathode featuring universal ion adaptability and suitable redox potential to realize an efficient all‐polymer full‐seawater battery, especially one that performs reliably at low temperatures, is still a challenging task owing to the limited choices of seawater‐compatible polymer electrodes which can maintain high redox activity in near‐neutral conditions.

In this research, we introduce for the first‐time a coupled n‐type/p‐type redox chemistry in natural seawater to establish a new class of all‐polymer rechargeable full‐seawater battery (APRSB), which addresses and overcomes several key limitations (Figure 1b) of conventional RSBs based on metal‐air chemistry to yield high energy efficiency, superior rate capability, and excellent cycling stability even under sub‐zero temperature conditions. In our approach, a flexible triamine linker was introduced to crosslink the naphthalenetetracarboxylic dianhydride (NTCDA) molecular assemblies into a naphthalenetetracarboxylic diimide polymer (NDIP, Figure 1c),^[^ ^25^, ^26^ ^]^ forming an amorphous polymer network that facilitates efficient charge carrier transport. With such an aggregate structure regulation, the newly‐designed n‐type redox NDIP is able to accommodate diverse cations (Na^+^, K^+^, Mg^2+^, Ca^2+^) and afford consistently excellent storage capability in stark contrast to the crystalline NTCDA counterpart with much inferior performance. This enables an efficient seawater‐adaptable low‐potential polymer anode with rapid redox kinetics and a high specific capacity of 138.1 mAh g^−1^ along with a long lifespan (84.2% retention over 10000 cycles)—outperforming most reported electrodes in seawater. To complement the NDIP anode, a p‐type high‐potential redox radical polymer (RRP, Figure 1d) is judiciously selected as a seawater‐compatible organic cathode, leveraging its intrinsic radical structure to ensure fast redox reactions and excellent compatibility with Cl^−^ and SO_4_^2−^ anions. As such, the combination of NDIP and RRP in natural seawater yields a novel all‐polymer seawater battery with an average output voltage of 1.2 V, a superior rate capability, a high energy efficiency of 81%, and a long cycle life over 800 cycles even at −4 °C—superior to the room‐temperature (RT) results of most reported seawater batteries involving air‐cathode chemistry. Additionally, the effects of low temperature on the organic redox kinetics of our APRSB system are investigated to further validate its performance under challenging environmental conditions.

## 2 Results and Discussion

**Figure 2:**
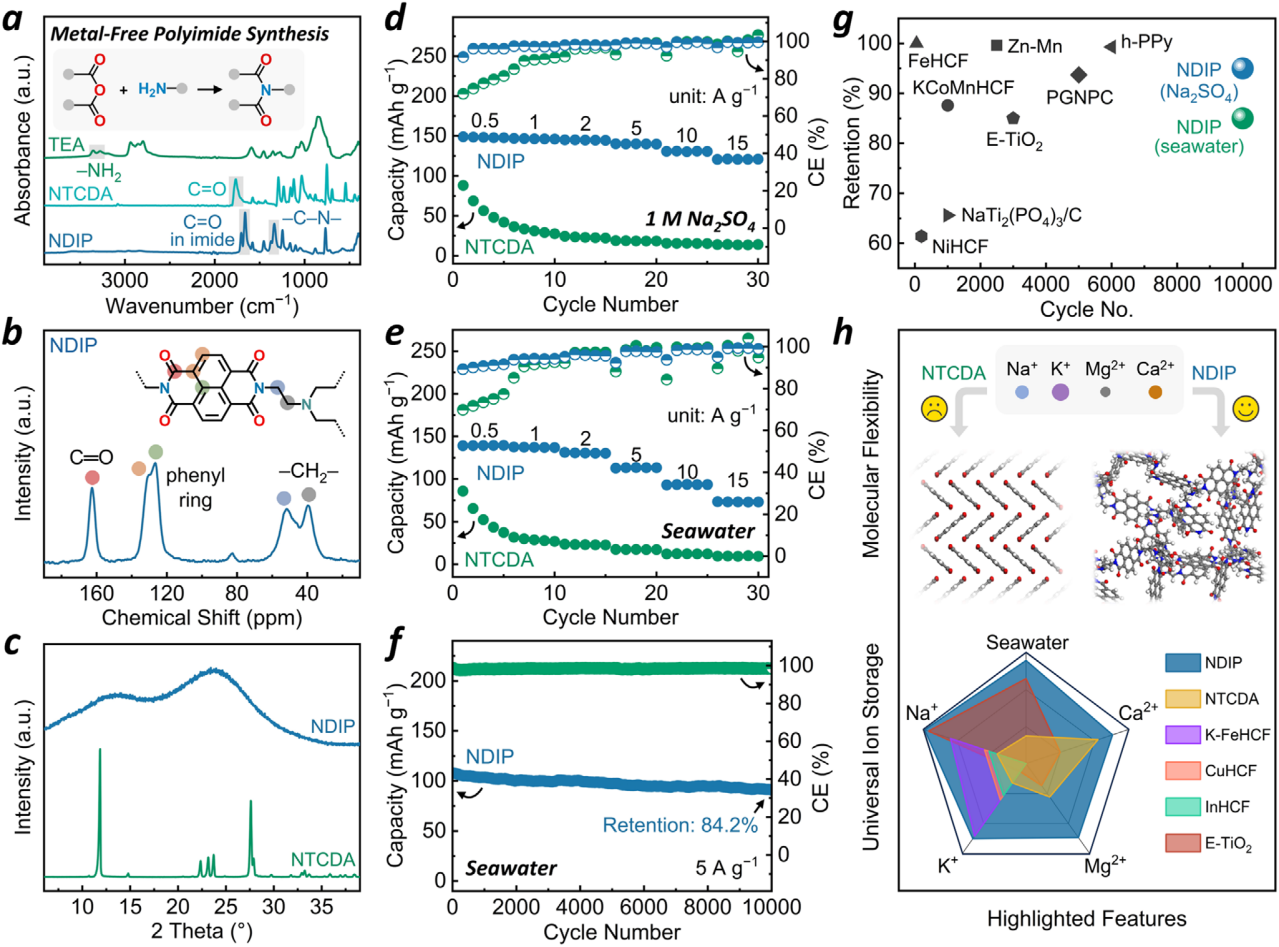
a) ATR‐FTIR spectra of TEA, NTCDA, and NDIP. Inset is the illustration of the polyimide condensation reaction. b) Solid‐state ^13^C NMR spectrum of NDIP. c) PXRD patterns of NTCDA and NDIP. Rate capability of NTCDA and NDIP in d) 1 M Na_2_SO_4_ and e) natural seawater. f) Cyclic stability of NDIP at 5 A g^−1^ in natural seawater. g) Capacity retention of NDIP and other electrodes for seawater applications. h) Schematic illustration of different‐cation storage capacities of NDIP, NTCDA and other reported inorganic electrodes for RSBs (full scale value: 150 mAh g^−1^).

To construct all‐polymer RSBs, the NDIP anode was first synthesized by coupling flexible tris(2‐aminoethyl)amine (TEA) linkers and the redox‐active 1,4,5,8‐naphthalenetetracarboxylic dianhydride (NTCDA) units through metal‐free one‐pot condensation reaction (**Figure** **2**a, inset). The attenuated total reflection Fourier transform infrared (ATR‐FTIR) spectra in Figure 2a reveal the absence of –NH_2_ and the presence of C─N in the NDIP, while the characteristic C═O and ─CH_2_─ bands are observed in the solid‐state ^13^C nuclear magnetic resonance (^13^C NMR) spectroscopy (Figure 2b). The successful formation of the TEA‐linked polyimide structure is further supported by X‐ray photoelectron spectroscopy (XPS), Raman inspection, and elemental analysis (Figures S1, S2, and Table S1, Supporting Information). Distinct from its crystalline NTCDA monomer featuring sharp peaks in the powder X‐ray diffraction (PXRD) pattern, NDIP presents broad bands originated from its amorphous polymeric network (Figure 2c). Accordingly, scanning electron microscope (SEM) images reveals that NTCDA comprises compact rod‐like crystal, while NDIP exhibits irregular aggregate morphology consisting of abundant wrinkles and ridges (Figure S3, Supporting Information), contributing to its hydrophilic feature (Figure S4, Supporting Information), which is favorable for aqueous energy storage applications. Though exhibiting limited surface area and porosity (Figure S5, Supporting Information), NDIP shows consistent insolubility in various solvents (Figure S6, Supporting Information), which ensure its outstanding durability for long‐term electrochemical cycling.

The cyclic voltammetry (CV) technique was first employed to investigate the storage behavior of the NDIP toward Na^+^ at RT, which accounts for the dominant proportion among all ionic species in natural seawater (30.6% of total salt content). In 1 M Na_2_SO_4_ electrolytes, crystalline NTCDA exhibits sharp redox peaks in the first cycle with large peak‐to‐peak separations (Figure S7a, Supporting Information); ΔE_1_ = 273 mV, ΔE_2_ = 281 mV, ΔE_3_ = 198 mV; probably owing to the relatively sluggish ion insertion/extraction in its crystalline lattice), and suffers a major capacity loss in subsequent scans. By contrast, amorphous NDIP displays two pairs of well‐defined broad peaks with clearly smaller peak‐to‐peak separation (Figure S7b, Supporting Information; ΔE_1_ = 128 mV, ΔE_2_ = 106 mV) corresponding to the redox transformation of C═O/C─O^−^ in the imide ring accompanied by the reversible Na^+^ uptake/release (Figure S8, Supporting Information). According to these CV curves, the capacitive contribution can be estimated based on the well‐known Dunn method,^[^ ^27^ ^]^ which yields a result of 95–97% from 1 to 3 mV s^−1^, indicating its primarily surface‐controlled redox behavior (Figure S9, Supporting Information). Besides, NDIP affords a high apparent ion diffusion coefficient of ≈2.57 × 10^−9^ cm^2^ s^−1^ as calculated from the galvanostatic intermittent titration (GITT) technique (Figure S10, Supporting Information). Accordingly, as indicated by the galvanostatic charge‐discharge (GCD) profiles (Figure 2d), NDIP delivers a RT capacity of 148.9 mAh g^−1^ at 0.5 A g^−1^ and retains 120.6 mAh g^−1^ even at 15 A g^−1^, along with an exceptional stability with 95.5% capacity retention over 10000 cycles (Figure S11, Supporting Information). In contrast, NTCDA features inferior coulombic efficiency (CE) and severe capacity decay at low current densities, along with the negligible capacities at high current densities of 10 and 15 A g^−1^. Surprisingly, when tested in natural seawater, NDIP retains well comparable electrochemical performance with respect to that in 1 M Na_2_SO_4_. Similarly, NDIP in seawater possesses two pairs of broad redox peaks within −0.8–0 V vs. Ag/AgCl associated with the carbonyl transformation (Figures S12 and S13, Supporting Information), along with dominant capacitive contributions up to 86–92% (Figure S14, Supporting Information) and an apparent ion diffusion coefficient of ≈1.75 × 10^−9^ cm^2^ s^−1^ (Figure S15, Supporting Information). In particular, NDIP still holds large RT capacities of 138.1 mAh g^−1^ at 0.5 A g^−1^ and 76.8 mAh g^−1^ even at 15 A g^−1^ in seawater, whereas NTCDA displays almost no capacity at 15 A g^−1^ (Figure 2e), underscoring the distinct advantage of the amorphous polymeric structure of NDIP. Importantly, a superior cyclability with 84.2 % retention after 10000 cycles is also attained for NDIP (Figure 2f; Figure S16, Supporting Information), which outperforms most previous electrodes in seawater‐based systems (Figure 2g; Table S4, Supporting Information).

Considering the significant variation in the proportion of different cations in natural seawater (Na^+^: 30.6%, Mg^2+^: 3.7%, Ca^2+^: 1.2%, and K^+^: 1.1%) and the possibility that the dominant Na^+^ might shield the interaction of other cations with redox polymers, we further tested the ionic storage capability of NDIP in NaCl, KCl, MgCl_2_, CaCl_2_, each with a uniform cation concentration of 1 M. All four CV profiles show relatively similar redox peaks (Figure S17, Supporting Information), and quite consistent capacities of ≈120–130 mAh g^−1^ are acquired at 0.5 A g^−1^ in four different electrolytes (Figures S18 and S19, Supporting Information), indicating the universal ionic adaptability of NDIP for multiple‐cation storage. This is in sharp contrast to the inferior compatibility of NTCDA toward different cations, showing rapid capacity decay after several cycles at low current density and almost no capacity at high rates. To sum up, different from the rigid crystalline structure of NTCDA molecules, the amorphous cross‐linked network induced by the flexible linker endows NDIP with sufficient voids to accommodate cations of varying sizes and charges, affording excellent charge storage capability toward each seawater‐relevant cation, which is superior to many reported inorganic electrodes only active for specific cations rather than all (Na^+^/Mg^2+^/Ca^2+^/K^+^, Figure 2h; Table S5, Supporting Information). Taken together, the above results imply that the complex ionic environment of real seawater has a minor effect on the electrochemical performance of NDIP anode, manifesting its adaptability for seawater.

**Figure 3:**
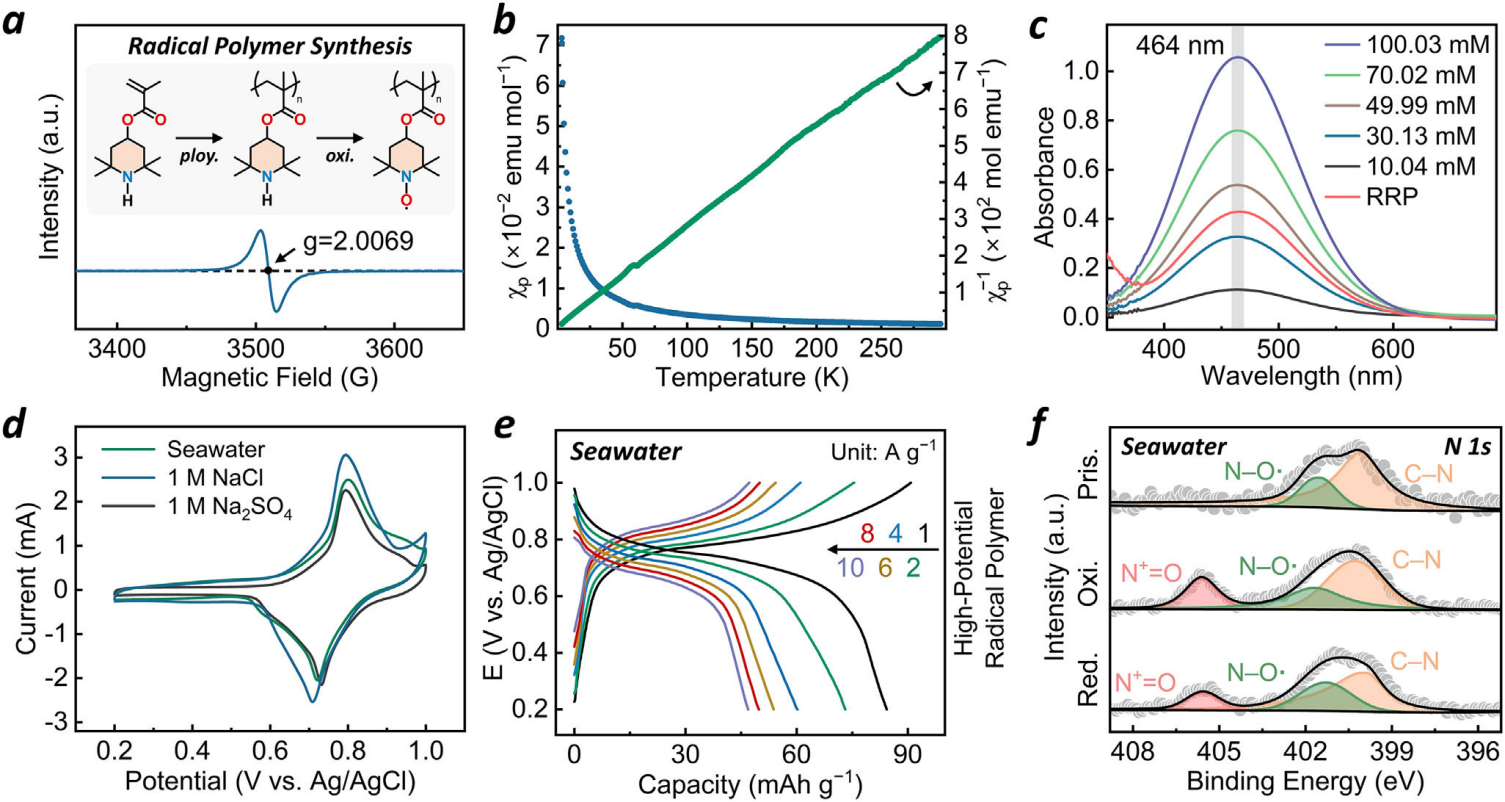
a) EPR spectrum of the RRP powder. Inset is the illustration of the pre‐polymerization (ploy.) and post‐oxidation (oxi.) synthetic procedure of RRP. b) Temperature paramagnetic susceptibility χ_p_ (blue plots) and its inverse χ_p_^−1^ (green plots) of RRP. c) UV spectrum of standard TEMPO‐OH series solution and RRP in dimethyl formamide. d) CV curves at 1 mV s^−1^ of RRP in 1 M Na_2_SO_4_, 1 M NaCl, and seawater. e) GCD profiles of RRP in seawater. f) High‐resolution of N 1s XPS spectra of RRP electrode at pristine, oxidized and reduced states in seawater.

In addition to developing low‐potential polymer anodes, identifying a well‐matched high‐potential polymer cathode remains a significant challenge for advancing APRSBs. Thus far, bifunctional oxygen‐involved catalysts and inorganic intercalation compounds have been extensively used as cathodes in RSBs, while the application of high‐potential organic cathode in this field remains scant. Herein, we synthesized a redox radical polymer (RRP) as a cathode to accommodate various anions in seawater. Specifically, we polymerize the 2,2,6,6‐tetramethylpiperidine methacrylate precursor using 2,2′‐azobisisobutyronitrile (AIBN) as the radical initiator, and the resulting polymer (Figure S20, Supporting Information) is further oxidized with H_2_O_2_ (catalyzed by ethylenediaminetetraacetic acid and Na_2_WO_4_·2H_2_O) to obtain the final RRP (**Figure** **3**a, inset; please see synthetic details in the Supporting Information). The weight average molecular weight (M_w_ = 553794) and number average molecular weight (M_n_ = 175969) of RRP were determined by size exclusion chromatography with DMF as the eluent, which gives a polydispersity of 3.15 and a degree of polymerization of 732 (Table S2, Supporting Information). As seen in Figure S21 (Supporting Information), several characteristic peaks (C─H at 2981 and 2947 cm^−1^, C═O at 1726 cm^−1^, and N–O• at 1363 cm^−1^) in the ATR‐FTIR spectra and the elemental analysis result (Table S3, Supporting Information) together demonstrate the successful production of radical polymer, which also exhibits an amorphous feature (Figure S22, Supporting Information). We further employed the electron paramagnetic resonance (EPR) spectroscopy to provide direct evidence of the formation of nitroxyl radical in RRP. Figure 3a shows the EPR spectrum of solid RRP powders with g = 2.0069 as a typical nitroxyl radical value.^[^ ^28^ ^]^ Moreover, the magnetic property of RRP was also studied by using a magnetometer to plot the temperature dependent paramagnetic susceptibility (χ_*p*_) within a temperature range 2–300 K (Figure 3b, blue plots). The linear fitting curve of the reciprocal of χ_*p*_ versus temperature (Figure 3b, green plots) implies the paramagnetic characteristics of RRP, while its radical concentration is computed to be 0.995 unpaired electron for each repeating unit. The ultraviolet‐visible (UV) spectroscopy was also applied to estimate the radical concentration, which yields a result of 0.898 unpaired electron per repeating unit (Figure 3c; Figure S23, Supporting Information). The variation between results derived from magnetic and spectroscopy tests is inevitable due to the inherent errors in these different techniques.^[^ ^29^ ^]^ In brief, the theoretical capacity of RRP is determined to be 100–110 mAh g^−1^ when assuming the radical concentration is in the range of 0.898–0.995 per unit. Based on the thermogravimetric analysis (TGA) curve in Figure S24 (Supporting Information), the obvious weight loss of the RRP powder occurs at a relatively high temperature of 250 °C (probably due to the rupture of the ester bond and the alkyl chain), suggesting that its thermal stability is adequate for practical aqueous battery applications.

Given that the main anions in seawater are Cl^−^ (55.0% of total salts) and SO_4_^2−^ (7.6%), the electrochemical performance of RRP was assessed in 1 M Na_2_SO_4_, 1 M NaCl, and natural seawater, respectively. Clearly, in three different electrolytes, RRP presents relatively consistent CV curves within 0.2–1.0 V vs. Ag/AgCl, featuring sharp and reversible redox peaks with small peak‐to‐peak separation (Figure 3d, ΔE < 80 mV, close to the theoretical value of 59 mV). In seawater, RRP shows a capacity of 84 mAh g^−1^ at 1 A g^−1^ (≈76% of its theoretical capacity of 111 mAh g^−1^, probably due to the completeness limit of the post‐oxidation reaction during the RRP preparation as mentioned above), which maintained 47 mAh g^−1^ at 10 A g^−1^ (Figure 3e; Figure S25, Supporting Information). RRP also presents a high apparent ion diffusion coefficient of ≈4.62 × 10^−9^ cm^2^ s^−1^ as computed from the GITT curves (Figure S26, Supporting Information). Such a superior rate capability and ionic transport can be ascribed to the facile p‐type radical redox process strongly linked with intrinsic feature of the radical, which involves minor structural and bond rearrangement,^[^ ^30^ ^]^ as validated in the ex‐situ XPS and EPR spectroscopy (Figure 3f; Figures S27 and S28, Supporting Information). Similar capacity and rate performance for RRP were also attained in 1 M Na_2_SO_4_ and 1 M NaCl electrolytes, indicating excellent compatibility of RRP with different anions (Cl^−^ and SO_4_^2−^) and its adaptability to the complex seawater environment (Figures S29 and S30, Supporting Information).

Taken together, our newly‐designed n‐type NDIP anode exhibits a low redox potential and excellent charge storage ability for multiple cations (Na^+^/K^+^/Mg^2+^/Ca^2+^), while the p‐type RRP cathode operates at a high redox potential and effectively accommodates both Cl^−^ and SO_4_^2−^ anions, making both electrodes well‐suited for natural seawater‐based energy storage and enabling the construction of an APRSB by pairing NDIP with RRP. Such a seawater battery is essentially based on a ready‐to‐charge dual‐ion mechanism, i.e., the assembled full battery needs to be charged first, wherein the NDIP anode is reduced to uptake cations and the RRP cathode is oxidized to store anions (Figure S31, Supporting Information); during discharge, NDIP and RRP gradually return to their neutral state and the cations/anions are released back to the electrolyte. Prior to assembly, we have confirmed the consistency of the ionic composition and pH for different batches of seawater (Table S6, Supporting Information). CV tests also indicate that NDIP and RRP show consistent performance in these seawater and their redox responses are mainly attributed to the dominant Na^+^ and Cl^−^ ions (Figures S32 and S33, Supporting Information). We also demonstrated that the redox potential range of NDIP is not negative enough to trigger hydrogen evolution reaction (HER), ensuring both the safety and efficiency of the APRSB (Figure S34, Supporting Information).

**Figure 4:**
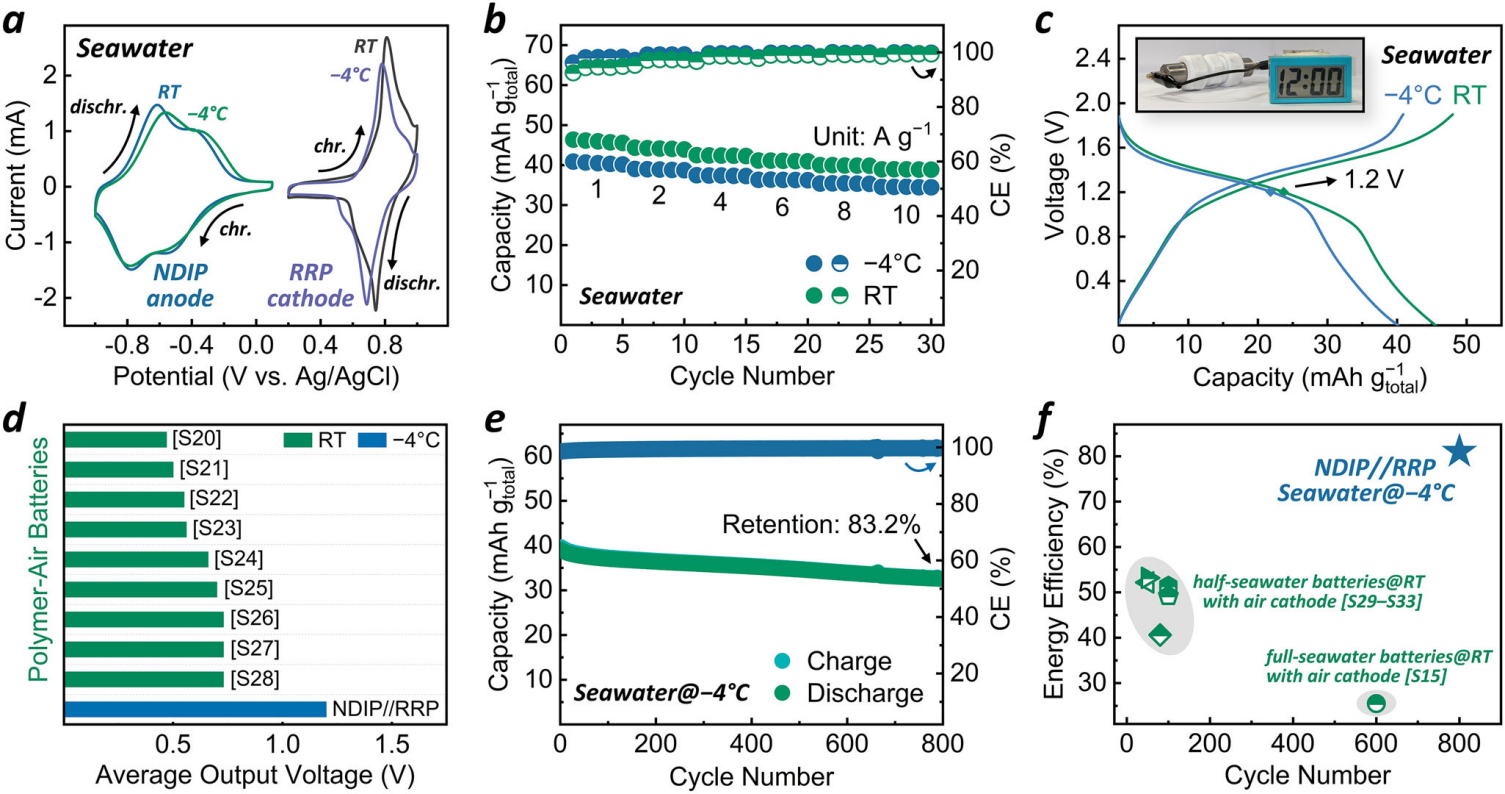
a) CV comparison at 1 mV s^−1^ of NDIP anode and RRP cathode in seawater at RT and −4 °C. b) Rate capability comparison and c) GCD profile comparison at 1 A g^−1^ of the NDIP//RRP cell at RT and −4 °C. Inset is a photo showing that one NDIP//RRP cell powers a digital clock at −4 °C. d) Average output voltage comparison with the previous RT aqueous polymer‐air batteries. e) Cycling stability of NDIP//RRP cell at −4 °C. f) Energy efficiency and cycle lifespan comparison with reported RT seawater batteries with air cathodes.

Finally, we assembled NASICON membrane‐free APRSB with NDIP, RRP, and natural seawater as the anode, cathode and electrolyte, respectively. Due to the Cl^−^ corrosion issue on coin cells made of stainless‐steel (Figure S35, Supporting Information), a two‐electrode Swagelok cell configuration is adopted for APRSB assembly. Considering the freezing point of our adopted seawater is around −6 °C (Figure S36, Supporting Information), we probed the low‐temperature operation feasibility of both polymer electrodes and APRSB in natural seawater at −4 °C. Interestingly, for either NDIP or RRP electrode, nearly overlapped CV profiles are observed at RT (≈20 °C) and −4 °C, indicative of quite similar redox activity (**Figure** **4a**). Similar capacities are also attained at two different temperatures, especially at low current densities (Figures S37 and S38, Supporting Information). These manifest that low temperature has minor effect on the organic redox reactions at both anode and cathode in our case. As anticipated, the assembled APRSB can work at −4 °C to produce a discharge capacity of 40.9 mAh g^−1^ at 1 A g^−1^ (Figure 4b; Figure S39, Supporting Information, based on the total mass of both anode and cathode active materials), and remain 34.5 mAh g^−1^ at 10 A g^−1^ (84.4% retention of that at 1 A g^−1^, indicative of high rate capability), on a par with its RT performance (46.4 and 38.9 mAh g^−1^ at 1 and 10 A g^−1^). Besides, the electrochemical impedance spectroscopy (EIS) results for APRSB are also comparable at −4 °C and RT (Figure S40, Supporting Information). Taken together, the above detailed comparative analyses verify the minute influence of variable temperature on organic redox kinetics. Notably, APRSB at −4 °C can maintain an average discharge voltage of 1.2 V—the same as the RT result (Figure 4c), superior to most reported aqueous polymer‐air batteries (typically ≤ 0.8 V, Figure 4d and Table S7, Supporting Information), and comparable to some primary seawater batteries (1–1.6 V) based on Mg/Al/Zn‐air chemistry.^[^ ^31^, ^32^ ^]^ Therefore, only one APRSB is capable of powering a digital clock at −4 °C (Figure 4c, inset), indicating its great potential for low‐temperature energy storage application. Impressively, the APRSB at −4 °C yields a high energy efficiency up to 81%, which could be attributed to coupled n‐/p‐type redox chemistry that promotes favorable organic redox kinetics despite low temperatures. Importantly, the APRSB at −4 °C attains a capacity retention of 83.2% after 800 cycles at 1 A g^−1^ (Figure 4e). Such high energy efficiency and cycling lifespan even exceed the RT results of many previous seawater batteries based on air cathodes (Figure 4f; Table S8, Supporting Information). Although the capacity of the APRSB is inherently limited by the theoretical capacity of redox polymers and differs in storage mechanism from conventional metal‐air seawater batteries, its performance is still competitive and even exceeds those of many existing all‐polymer aqueous batteries.^[^ ^33^, ^34^, ^35^, ^36^ ^]^ Nevertheless, this is the first report on all‐polymer full‐seawater batteries and a successful attempt of combined n‐/p‐type organic redox chemistry for sustainable low‐temperature seawater energy storage, avoiding complex cell configuration, the dendrite issue of Na anodes, and Cl^−^ side effects on cathodes that plague traditional RSBs, which also offers appealing potential for flexible devices (Figure S41, Supporting Information). Our APRSB delivers a high energy efficiency, rate capability, and cycling stability even at low temperature, opening a promising avenue toward the RSB development. For future applications, further enhancing the energy density in the device scale and comprehensively understanding the storage mechanism are major challenges, which are still underway.

## 3 Conclusion

In summary, a coupled n‐type/p‐type redox chemistry instead of traditional metal‐air chemistry has for the first time been proposed to develop a new family of all‐polymer rechargeable full‐seawater battery, displaying high energy efficiency and cycling stability even at low temperature. Our strategy involves the rational design and synthesis of two well‐matched seawater‐adaptable redox polymers: an n‐type NDIP anode and a p‐type RRP cathode. By using flexible triamine linker to cross‐link redox‐active NTCDA molecular assemblies for facilitated access of electrolyte and charge carriers via aggregate structure regulation, the newly‐acquired NDIP affords excellent storage capability for multiple cations (Na^+^/Mg^2+^/Ca^2+^/K^+^) with consistently high capacity, in sharp contrast to NTCDA with rather poor performance. This enables an efficient low‐potential polymer anode with fast redox kinetics and large specific capacity of 138.1 mAh g^−1^ along with superior cyclability over 10 000 cycles in seawater—surpassing most literature results in seawater‐based electrolytes. A p‐type RRP is explored as a high‐potential seawater‐suitable cathode to well match the NDIP anode, which combines excellent adaptability for both Cl^−^ and SO_4_^2−^ anions and facile radical redox property in seawater. Thus, pairing NDIP and RRP in natural seawater enables a brand‐new all‐polymer seawater battery with an average output voltage of 1.2 V, superior rate capability, high energy efficiency of 81%, and a long cycle life over 800 cycles even at −4 °C—exceeding RT results of most existing seawater batteries based on air‐cathode chemistry. Detailed studies unveil facilitated organic redox reaction kinetics resistive against low temperature in seawater. Our findings not only highlight a novel class of redox polymer electrodes and all‐polymer rechargeable seawater batteries but also render a fresh design paradigm of next‐generation seawater systems for sustainable low‐temperature energy storage.

## Conflict of Interest

The authors declare no conflict of interest.

## Supporting information



Supporting Information

## Data Availability

The data that support the findings of this study are available in the supplementary material of this article.
