# Supplementary material for: Beyond Metal‐Air Chemistry: Coupling n‐/p‐Type Organic Redox Chemistry Toward Sustainable Sub‐Zero‐Temperature All‐Polymer Seawater Batteries
Source: Adv Sci (Weinh). 2025 Aug 4;12(40):e04004. doi: 10.1002/advs.202504004 (PMC12561187; doi:10.1002/advs.202504004)
Supplement: Supplementary file 1 — Supporting Information [file ADVS-12-e04004-s001.pdf]

Supporting Information

Beyond Metal-Air Chemistry: Coupling n-/p-Type Organic Redox Chemistry Toward Sustainable Sub-Zero-Temperature All-Polymer Seawater Batteries

Yuanzhe Lu,[†1] Xiu Liu,[†1] Fan Yang, [†1] Zishou Zhang,[1] Fei Xin,*[2] Linfeng Zhong,*[1] and Dingshan Yu*[1]

[1]Key Laboratory for Polymeric Composite and Functional Materials of Ministry of Education, Key Laboratory of High-Performance Polymer-based Composites of Guangdong Province, GBRCE for Functional Molecular Engineering, School of Chemistry, School of Chemical Engineering and Technology, Sun Yat-sen University Guangzhou 510006, China

[2]School of Light Industry Science and Engineering, Beijing Technology and Business University

[†]These authors contributed equally to this work.

*Corresponding author: Fei Xin

E-mail: xinfei@th.btbu.edu.cn

*Corresponding author: Linfeng Zhong

E-mail: zhonglf27@mail.sysu.edu.cn

*Corresponding author: Dingshan Yu

Email: yudings@mail.sysu.edu.cn

Chemicals and Reagents

The reagents and compounds used in this research were purchased from commercial sources without further purification.

Precursors. Tris(aminoethyl)amine (TEA), mesitylene (MES) and isoquinoline (ISO) were acquired from Energy Chemical. 1,4,5,8-naphthalenetetracarboxylic dianhydride (NTCDA) was obtained from J&K Scientific. 2,2'-azobisisobutyronitrile (AIBN) was purchased from Aladdin. 2,2,6,6-tetramethylpiperidine methacrylate (TMPM) was purchased from TCI.

Electrolytes. Sodium sulfate (Na_2SO_4) was obtained from Aladdin. Sodium Chloride (NaCl) was purchased from Guangzhou Guanghua Sci-Tech CO. Ltd. Magnesium chloride ($MgCl_2$) was acquired from Tianjin Yongda Chemical Reagent Company Limited. Potassium chloride (KCl) was purchased from Heowns. Calcium chloride ($CaCl_2$) was obtained from Guangzhou Chemical Reagent Factory. Seawater was collected in Lingshan Bay, Qingdao, China.

Auxiliary. N, N-dimethylformamide (DMF) was obtained from Aladdin. N-methyl-2-pyrrolidone (NMP) was purchased from J&K Scientific. Tetrahydrofuran (THF) was acquired from Macklin. Carbon cloth was obtained from CeTech Co., Ltd. We used Ketjen Black (KB) as a conductive agent and polyvinylidene fluoride (PVDF) as a binder.

Characterizations

The solid-state ^{13}C nuclear magnetic resonance (^{13}C NMR) spectra were acquired on a Bruker Avance Neo 400WB spectrometer. The attenuated total reflection Fourier-transform infrared (ATR-FTIR) spectra were obtained on a Thermo Nicolet iS50 spectrometer. The powder X-ray diffraction (PXRD) patterns were collected on a Rigaku Smartlab (Cu Kα radiation, $\lambda = 1.5418$ Å, 40 kV, scan rate: 10° per minute). Cold field emission scanning electron microscopy (SEM) images were obtained using a Hitachi S-4800 equipment. Thermogravimetric analysis (TGA) was performed on a NETZSCH TG 209F1 Libra thermogravimetric analyzer. X-ray photoelectron spectroscopy (XPS) was conducted on a Thermo Scientific K-Alpha with a Al Kα radiation at 1486.6 eV, using the C1s = 284.8 eV to calibrate the binding energies of all elements. The Raman spectra were recorded with a Raman microscope (Renishaw, inVia Qontor) with a 325 nm laser as excitation source. The contact angle test was performed using a Chengde Dingsheng JY-82C video contact angle tester. The element analysis (EA) was done on Elementar Vario EL Cube CHNS/O elemental analyzer. The real density data of NDIP was obtained from

Micromeritics AccuPyc II 1340 for diffusion coefficient calculations. The UV-Vis spectra were obtained on a PerkinElmer UV750 spectrometer. The magnetic properties of RRP were studied by an electron spin resonance spectrometer (Bruker EMXplus) and a physical property measurement system (PPMS, MPMS XL-7). The composition of seawater was analyzed using ion chromatography (Thermo Scientific Dionex ICS-5000 and ICS-900 instruments). The molecular weight of RRP was determined by gel permeation chromatography (GPC, Waters 515 HPLC pump system). Nitrogen sorption isotherms for NDIP were measured on a Micromeritics ASAP 2460 instrument, with the specific surface area calculated using the Brunauer–Emmett–Teller (BET) method. Gas chromatography spectra were recorded on a SHIMADZU GC-2014 gas chromatograph.

Synthetic Process

Synthesis of NDIP. Tris(aminoethyl)amine (300mg, 2.05 mmol) and naphthalenetetracarboxylic dianhydride (NTCDA, 825.3mg 3.08 mmol) were placed in a 120 mL thick-walled bottle containing 20 mL of mesitylene (MES) and 20 mL of N-methyl-2-pyrrolidinone (NMP), followed by 10 minutes of sonication to obtain a homogenous mixture. After that, the bottle was frozen by liquid nitrogen for 5 minutes. Subsequently, when 2 mL of isoquinoline was added, and the whole bottle was vacuumized, purged with Argon gas, and then heated to 180 °C for 72 hours. We use vacuum filtration to collect the precipitate, which was washed by methanol, tetrahydrofuran (THF), N, N-dimethylformamide (DMF), and deionized water. After overnight drying at 70 °C, a dark brown powder was produced as the NDIP anode active materials.

Synthesis of RRP. The 2,2,6,6-tetramethylpiperidine methacrylate (TMPM) monomer (2.25g, 10 mmol) and 2,2'-azobisisobutyronitrile (AIBN, 0.04 g, 0.05 mmol) were dissolved in 5 mL of methanol. The mixture was stirred at 70 °C for 12 hours under N_2 atmosphere, then put into 50 mL of hexane and stirred for 5 min. The product (PMTMP) was obtained by centrifugation. Then, $Na_2WO_4 \cdot 2H_2O$ (0.49 g, 1.5 mmol), ethylenediaminetetraacetic acid (EDTA, 0.26 g, 0.9 mmol) and 30% H_2O_2 (8 mL) were added to the mixture comprising PMTMP (1.35 g, 6.0mmol), 40 mL methanol and 20mL H_2O, and the mixture was stirred at 60 °C for 48 hours. The resultant was filtrated and washed with H_2O and methanol to finally afford RRP as a pale red solid.

Theoretical Calculations

The geometry optimizations of NDIP and RRP fragments were performed on a Gaussian 09 software with B3LYP hybrid functional and 6-31G(d) basic set. The Multiwfn 3.8 program and Visual Molecular Dynamics (VMD) software was then applied to calculate and present the molecular electrostatic potential (MESP) results, respectively.

Electrochemical Characterization

NDIP-based electrode fabrication. 20 mg of PVDF was added into 1600 μL of NMP and stirred for 1 hour. 120 mg of NDIP and 60 mg of Ketjen Black (KB) were mixed using a ball milling apparatus for 30 minutes. Subsequently, the powder mixture was added into the aforementioned solution and stirred for another 6 hours. The slurry was spread onto a carbon cloth disk (12 mm in diameter), and then dried in a thermostatic oven at 70 °C overnight. The mass loading of NDIP on each carbon cloth disk is 1–1.4 mg cm^{-2}.

RRP-based electrode fabrication. 20 mg of PVDF was added into 1600 μL of NMP and stirred for 1 hour. A mixture of 100 mg of RRP and 80 mg of KB was subjected to ball milling for 30 minutes. Subsequently, the resultant was added into the aforementioned NMP solution and stirred for another 6 hours. The slurry was then spread onto a carbon cloth disk (12 mm in diameter), and then dried in a thermostatic oven at 70 °C overnight. The mass loading of RRP is 1–1.2 mg cm^{-2}.

Electrochemical equipment. All electrochemical measurements were conducted using CHI760E electrochemistry workstations or LAND CT3002A testing equipment.

Assembly of half cells. A conventional three-electrode setup was utilized to assess the electrochemical performance of a single electrode, consisting of a Ag/AgCl reference electrode, a platinum foil counter electrode, and the electrode under investigation as the working electrode. 1 M Na_2SO_4, seawater, 1 M NaCl, 1 M $MgCl_2$, 1 M KCl, and 1 M $CaCl_2$ were employed as electrolytes, respectively. Before test, the NDIP and RRP electrodes underwent five GCD cycles at a current density of 0.5 A g^{-1} and 1 A g^{-1} for activation, respectively.

Assembly of full cells. The full battery is assembled in the form of Swagelok cell, using NDIP anode and RRP cathode with a N/P ratio close to 1. Prior to assembly, the electrodes underwent five GCD cycles for activation (0.5 A g^{-1} for NDIP and 1 A g^{-1} for RRP).

Calculations of the electrochemical parameters.

The specific capacity (C_{sp}, mAh g^{-1}) is determined using the equation (1):

$$C_{sp} = \frac{I\,\Delta t}{3.6 \times m} \tag{1}$$

Here, I is the applied current density (mA), Δt represents the time interval (s), while m is the mass of active material (mg).

The theoretical capacity (C_{theo}, mAh g^{-1}) is computed according to equation (2):

$$C_{theo} = \frac{nF}{3.6 \times M} \tag{2}$$

Here, n represents the number of transferred electrons per repeating unit, F symbolizes the Faraday constant, while M signifies the molecular mass of the repeating unit (g mol^{-1}).

The b-value was calculated based on equation (3) and (4)

$$i_p = av^b \tag{3}$$

$$\log(i_p) = \log(a) + blog(v) \tag{4}$$

Equation (4) was derived from Formula (3) using logarithmic transformation, where b represents the slope of the fitted line, i_p indicates the peak current (mA) of cathodic or anodic peaks, v is the speed of scanning (mV s^{-1}), while a designates a constant.

Additionally, the quantitative analysis of diffusion-controlled contribution and pseudo-capacitive contribution can be achieved by equation (5).

$$i = k_1 v + k_2 v^{0.5} \tag{5}$$

In the given formula, i is the current (mA), $k_1 v$ represents the pseudo-capacitive contribution, while $k_2 v^{0.5}$ refers to the diffusion-controlled contribution part to the total current.

The ion diffusion coefficient is determined based on formula (6)

$$D_{ion} = \frac{4}{\pi t}\left(\frac{n_{mol} V_{mol}}{S_i}\right)^2 \left(\frac{\Delta E_s}{\Delta E_t}\right)^2 \tag{6}$$

Here, t denotes the pulse duration (s), n_{mol} represents the molar quantity of the active substance (mol), V_{mol} stands for the molar volume of the active substance (cm^3 mol^{-1}), S_i refers to the contact area (cm^2) between electrode disk and electrolyte, the potential difference before and after the current pulse is denoted as ΔE_s, while ΔE_t signifies the instantaneous potential difference at the start and the end of the current pulse.

The specific energy (E_{sp}, Wh kg^{-1}) is calculated based on formula (7):

$$E_{sp} = \frac{I \int U(t)dt}{3.6 \times m} \tag{7}$$

Here, the integral area of $U(t)$ over time is equal to $\int U(t)dt$, the applied current is labeled as I (mA), m refers to as the mass loading of active substance (mg).

The specific power (P_{sp}, W kg^{-1}) is determined by formula (8):

$$P_{sp} = \frac{3600 \times E_{sp}}{\Delta t} \tag{8}$$

Specifically, E_{sp} stands for specific energy (Wh kg^{-1}), Δt represents the variation of time (s).

The average output voltage (U_{ave}, V) is determined by formula (9):

$$U_{ave} = \frac{E_{sp}}{C_{sp}} \tag{9}$$

Literally, the specific capacity (mAh g^{-1}) is denoted as C_{sp}.

The energy efficiency (η_{VE}, %) is calculated based on formula (10):

$$\eta_{VE} = \frac{E_{sp-dis}}{E_{sp-ch}} \tag{10}$$

Here, E_{sp-dis} denotes the specific energy of the discharge process (Wh kg^{-1}), E_{sp-ch} represents the specific energy of the charge process (Wh kg^{-1}).

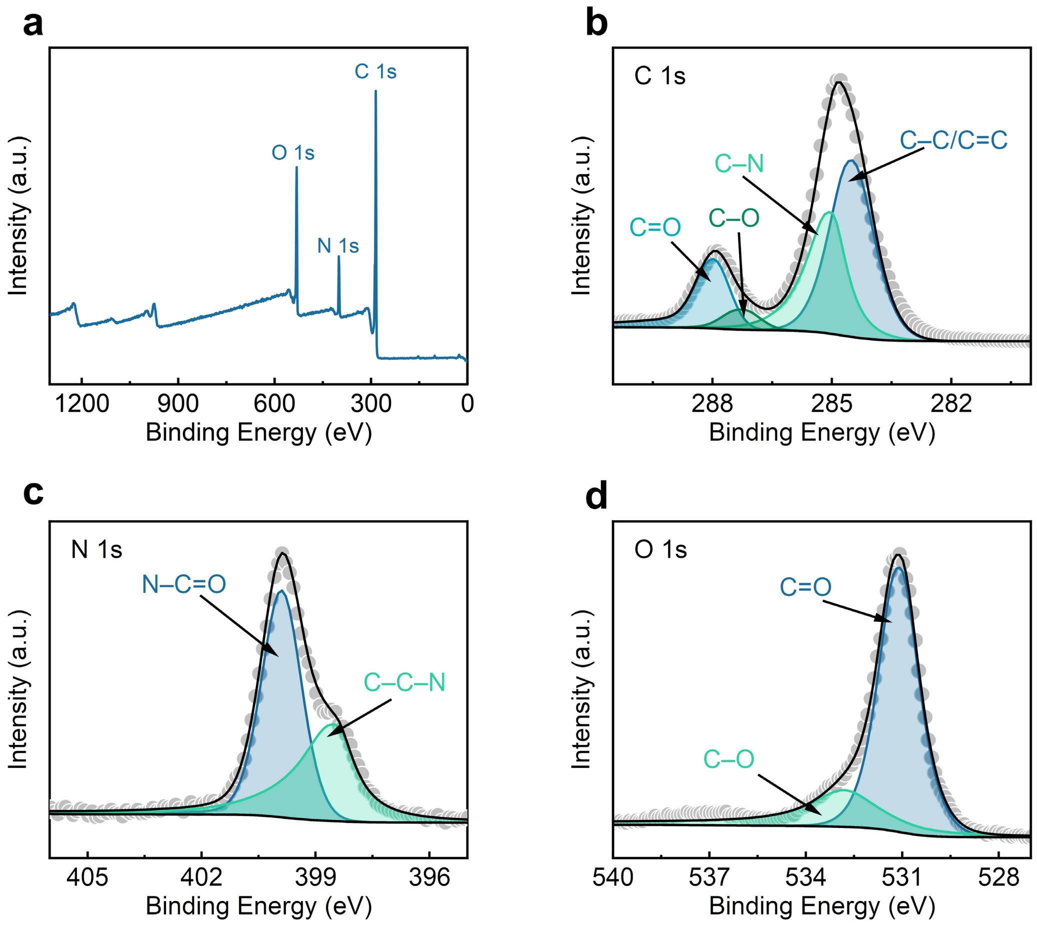

Figure S1. (a). The XPS full spectrum of NDIP. The high-resolution of (b) C 1s, (c) N 1s and (d) O 1s spectrum of NDIP.

Note： XPS spectroscopy provides an in-depth analysis of the chemical bonding components of NDIP. As can be seen in **Figure S1a**, the peaks of C, N and O confirmed the main elemental composition of NDIP. In **Figure S1b**, the peak at 285.1 eV indicates the presence of C–N, whereas the peaks at 287.3 eV and 288.0 eV correspond to C–O/C=O. In **Figure S1c**, the peaks at 399.9 eV and 398.6 eV in the N 1s profile are ascribed to N–C=O and C–C–N, respectively. Besides, in **Figure S1d** (O 1s spectrum), the signals at 531.3 eV and 532.9 eV can be ascribed to C=O and C–O, respectively. All these results validate the typical polyimide structure of NDIP.[S1]

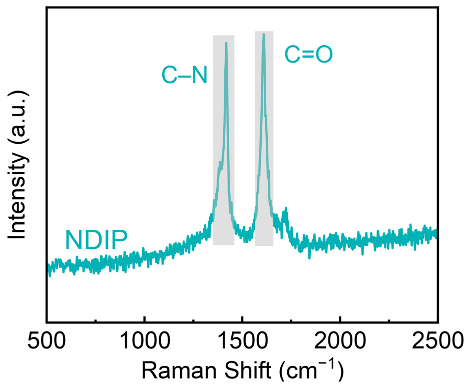

Figure S2. The Raman spectroscopy of NDIP powder.

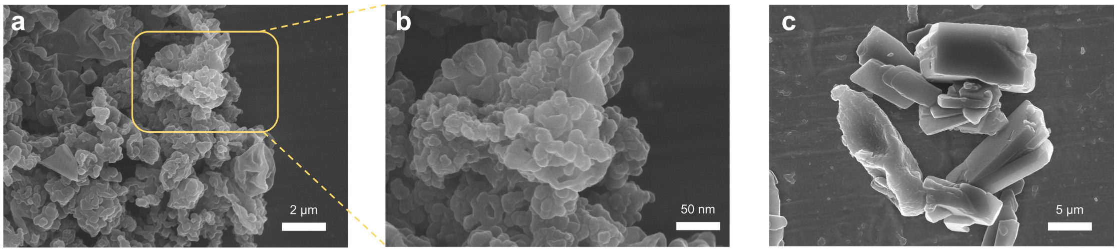

Figure S3. SEM images of (a-b) NDIP and (c) NTCDA.

Note: The amorphous feature of NDIP is further confirmed by SEM. Distinct from the rod-like morphology of NTCDA, NDIP exhibits amorphous aggregation of abundant nanoparticles, which might enhance its affinity to seawater electrolytes.

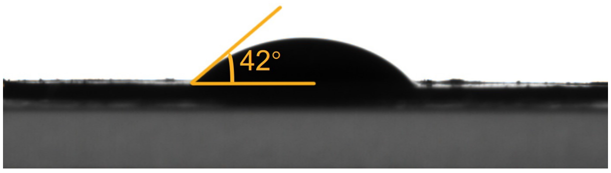

Figure S4. The contact angle between a drop of water and the pellet of NDIP powder.

Note: The hydrophilicity of NDIP in aqueous electrolyte is evidenced by the contact angle test, revealing a contact angle of 42° between a water droplet and the pellet pf NDIP particle.

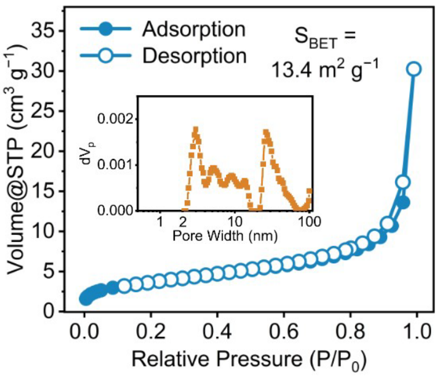

Figure S5. N_2 sorption isotherms and the pore size distribution (inset) of NDIP.

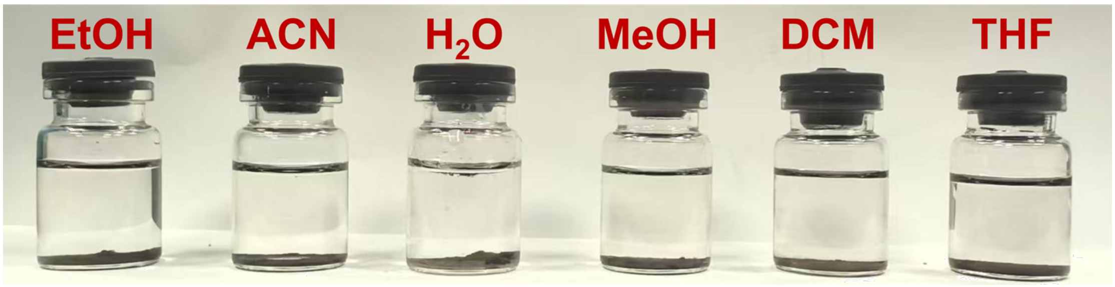

Figure S6. The insolubility of NDIP in various common solvents.

Note: EtOH: ethanol; ACN: acetonitrile; MeOH: methanol; DCM: dichloromethane; THF: tetrahydrofuran

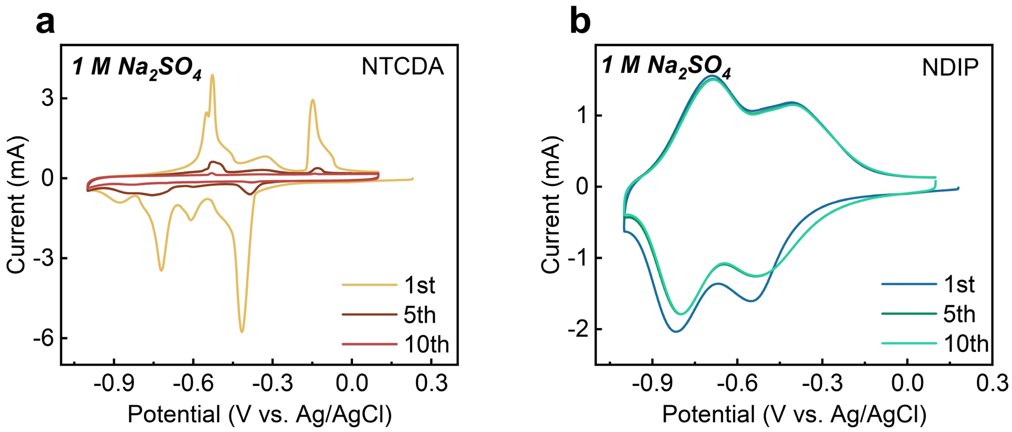

Figure S7. The CV curves of (a) NTCDA and (b) NDIP at different cycles in 1 M Na_2SO_4.

Note: The intensity of anodic/cathodic peaks of NTCDA in 1 M Na_2SO_4 reveals a significant decrease in 10 cycles (**Figure S7a**). In contrast, the curves of NDIP in 1 M Na_2SO_4 retain nearly identical after several cycles (**Figure S7b**), validating its storage stability towards Na^+ ions.

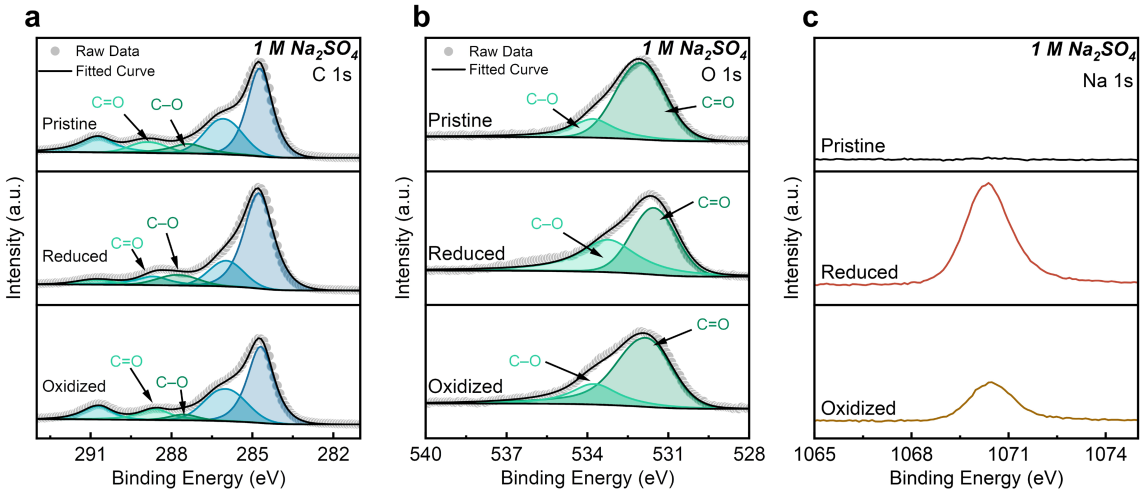

Figure S8. High-resolution of (a) C 1s (b) O 1s and (c) Na 1s XPS spectra of NDIP-based electrode at pristine, reduced and oxidized states in 1 M Na_2SO_4.

Note: These XPS spectra reveal the reversible transformation of C–O/C=O along with the Na^+ uptake and release, which validates the ion-coordination mechanism of NDIP.[S2]

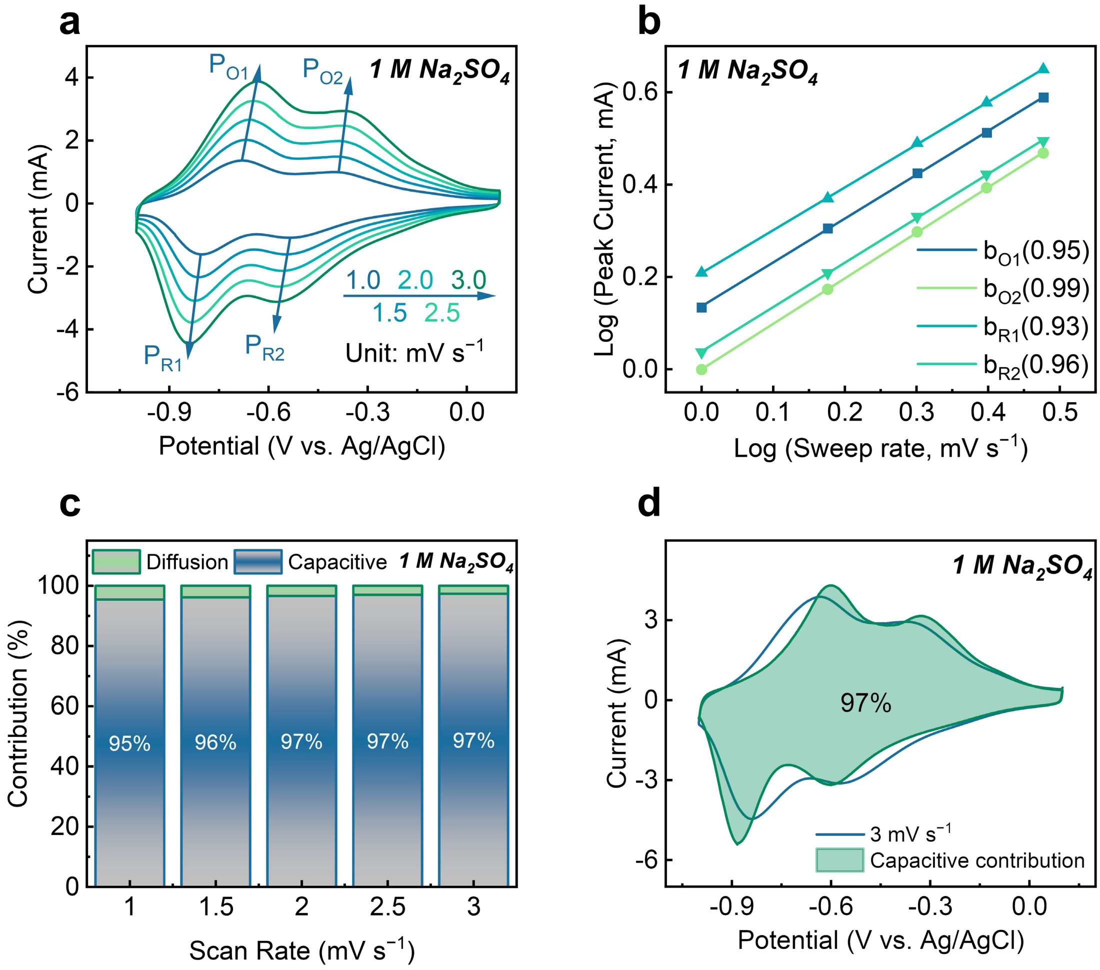

Figure S9. The results of (a) CV curves at different scan rates; (b) fitted lines for b-value; (c) column diagrams for capacitive contribution and (d) integral curve at 3 mV^{-1} of NDIP in 1 M Na_2SO_4.

Note: The kinetics analysis denotes that the electrode reaction is significantly controlled by pseudo-capacitive process.[S3]

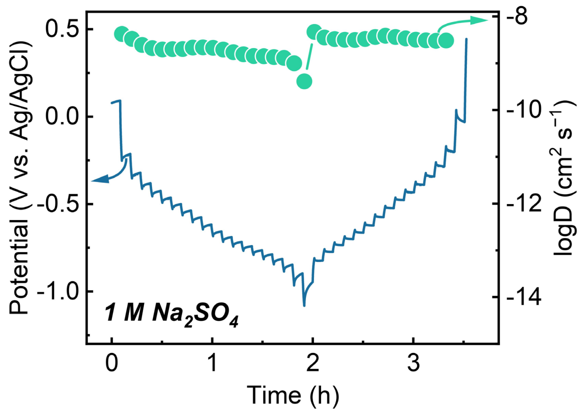

Figure S10. The GITT curves of NDIP in 1 M Na_2SO_4.

Note: The diffusion coefficient of Na^+ in 1 M Na_2SO_4 is around 2.57×10^{-9} cm^2 s^{-1}, which could be attributed to the amorphous structure of NDIP providing sufficient ion transportation channels.[S4]

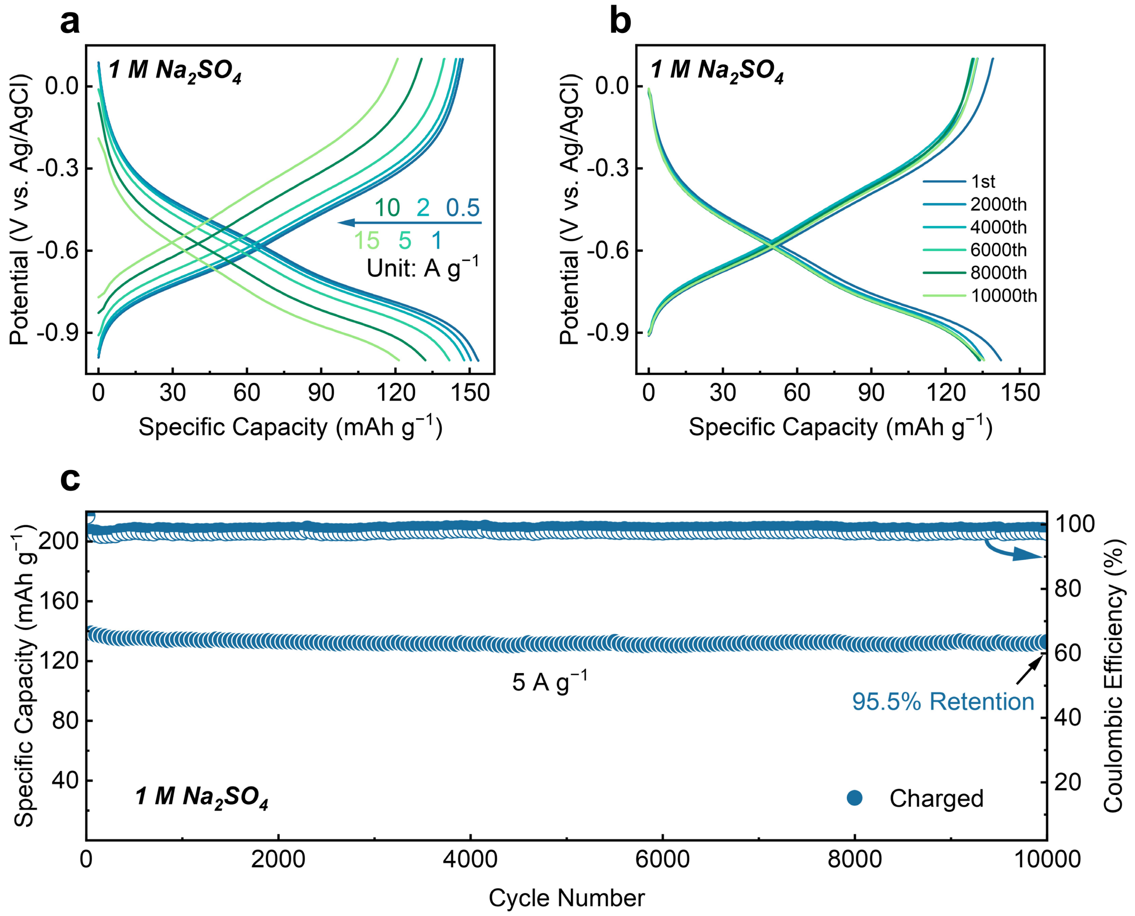

Figure S11. (a) GCD curves of NDIP at different current densities. (b) GCD curves of NDIP at different cycles in 1 M Na_2SO_4. (c) Cyclic stability of NDIP at the current density of 5 A g^{-1} in 1 M Na_2SO_4.

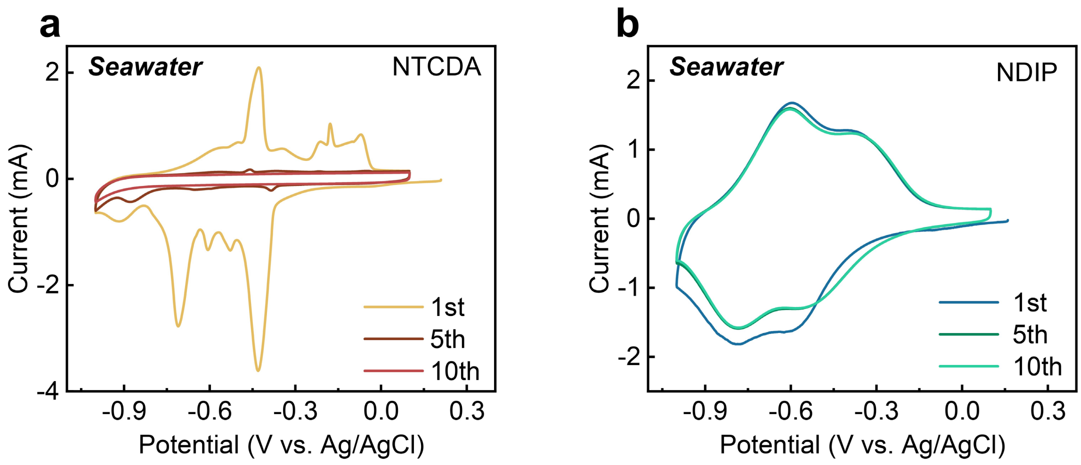

Figure S12. The CV curves of (a) NTCDA and (b) NDIP at different cycles in seawater.

Note: The intensity of anodic and cathodic peaks for NTCDA in seawater significantly diminishes following 10 cycles, while the curves of NDIP in seawater remain basically identical after 10 cycles, demonstrating its unique adaptability of NDIP for seawater.

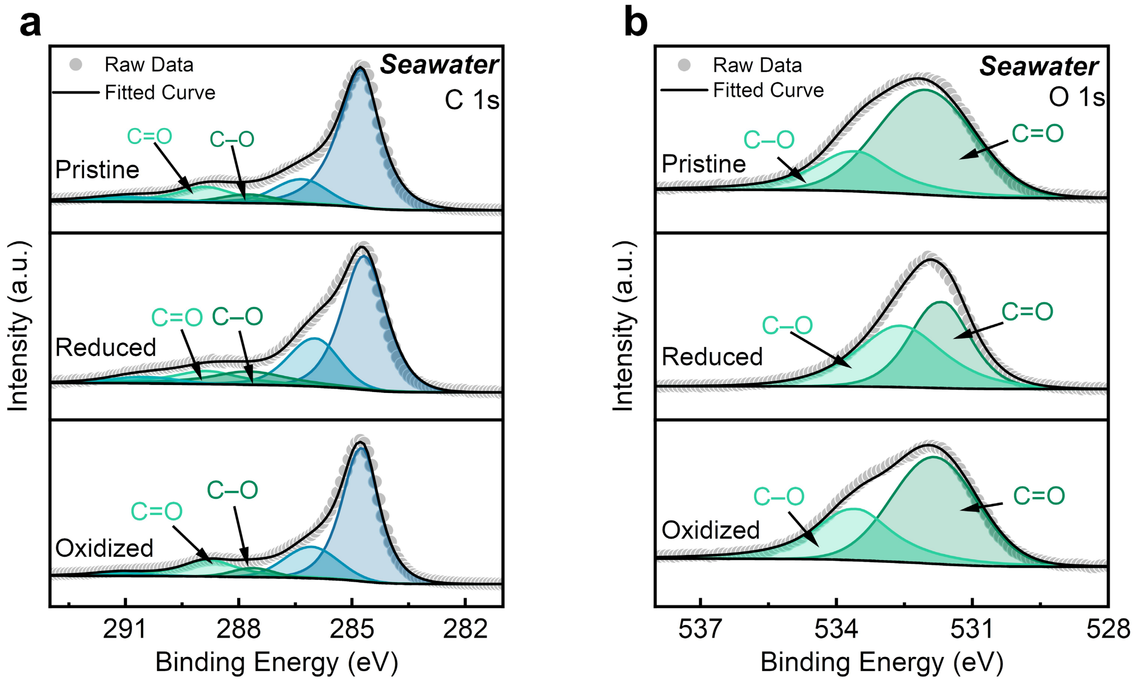

Figure S13. High-resolution of (a) C 1s and (b) O 1s XPS spectra of NDIP-based electrode at pristine, reduced and oxidized states in seawater.

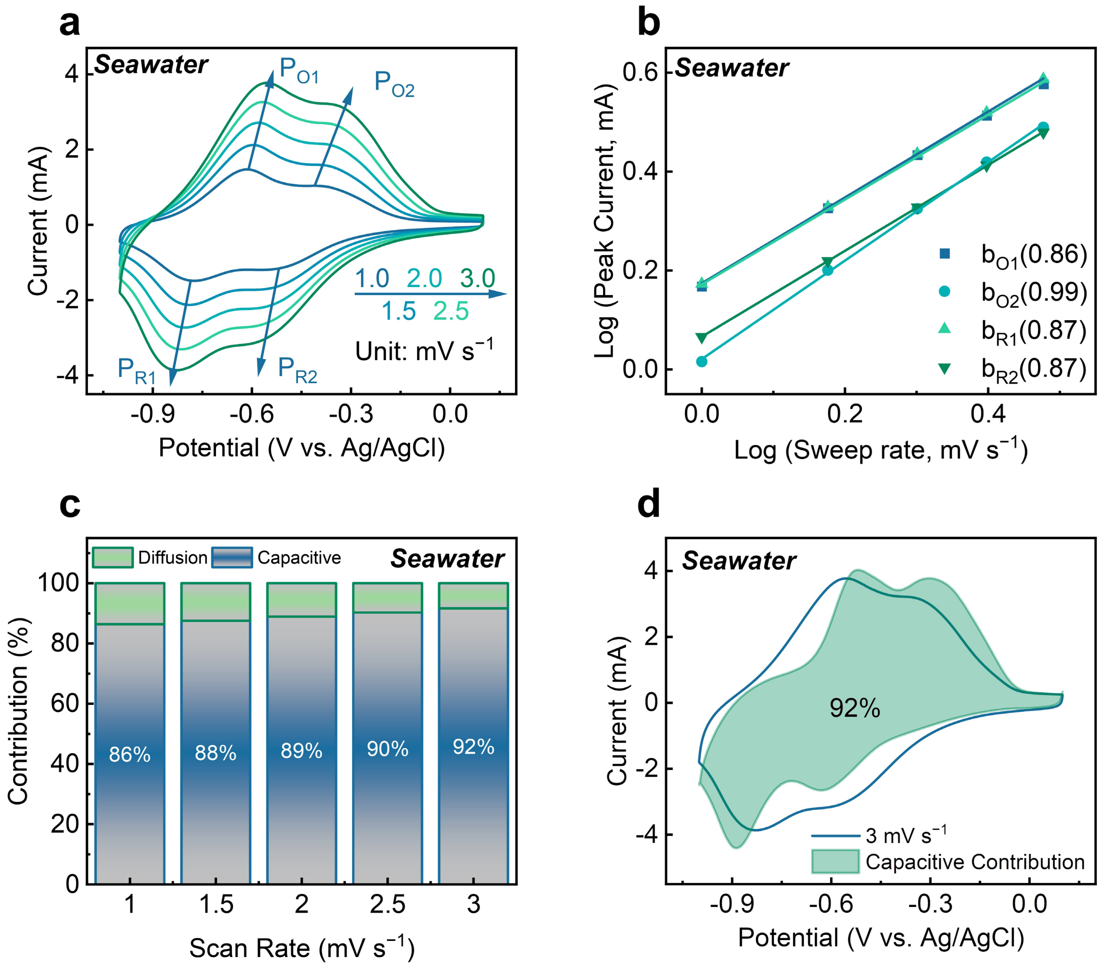

Figure S14. The results of (a) CV curves at different scan rates; (b) fitted lines for b-value; (c) column diagrams for capacitive contribution and (d) integral curve at 3 mV^{-1} of NDIP in seawater.

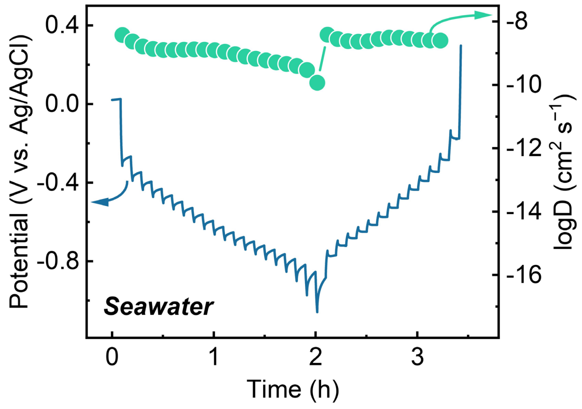

Figure S15. The GITT curves and the corresponding log(D) plots of NDIP in seawater.

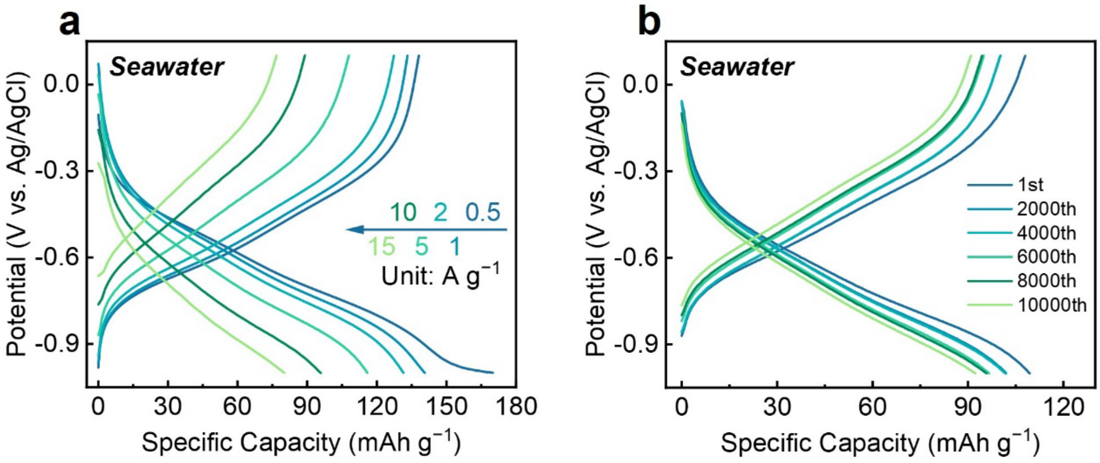

Figure S16. (a) GCD curves of NDIP at different current densities in seawater. (b) GCD curves of NDIP at different cycles in seawater.

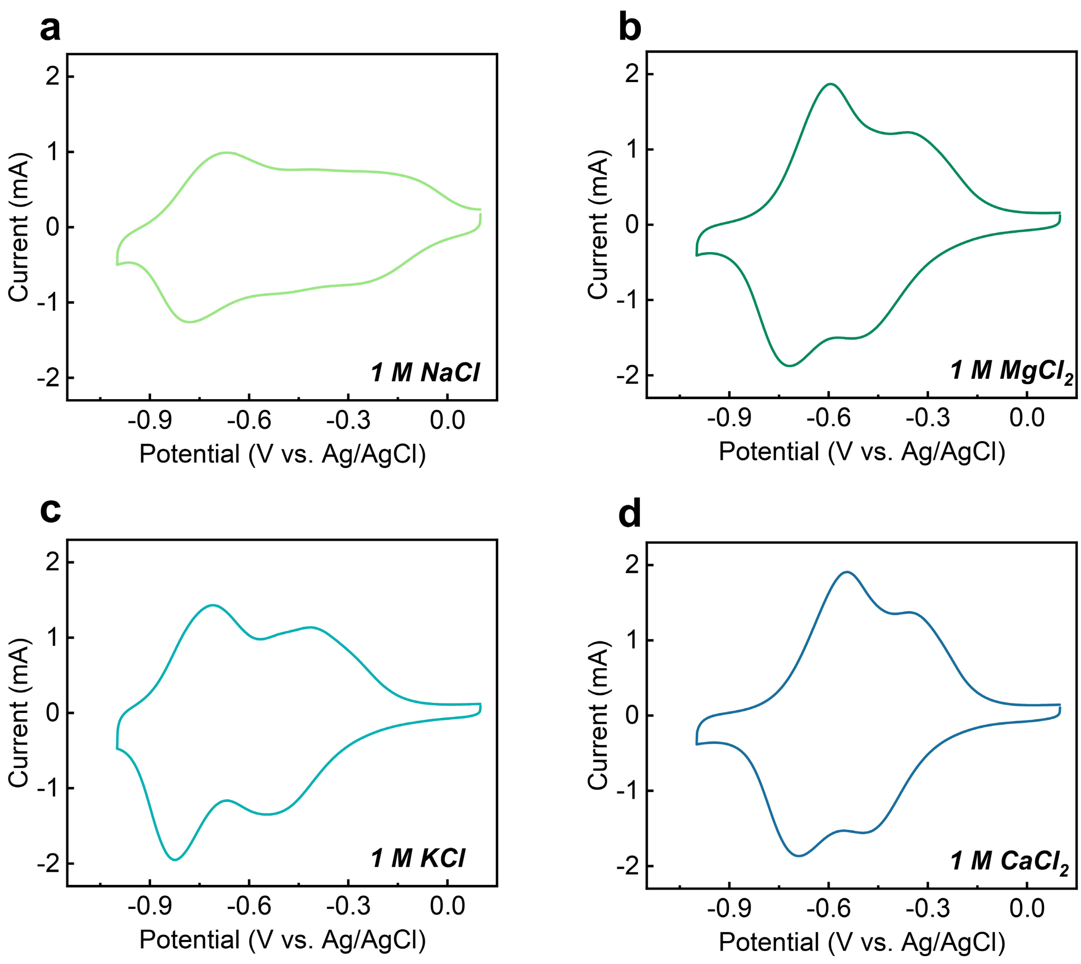

Figure S17. CV curves of NDIP in (a) 1 M NaCl, (b) 1 M $MgCl_2$, (c) 1 M KCl and (d) 1 M $CaCl_2$.

Note: The prominent redox peaks in all these electrolytes indicate that NDIP can interact with Na^+, Mg^{2+}, K^+, and Ca^{2+}, validating its unique universal cationic adaptability.

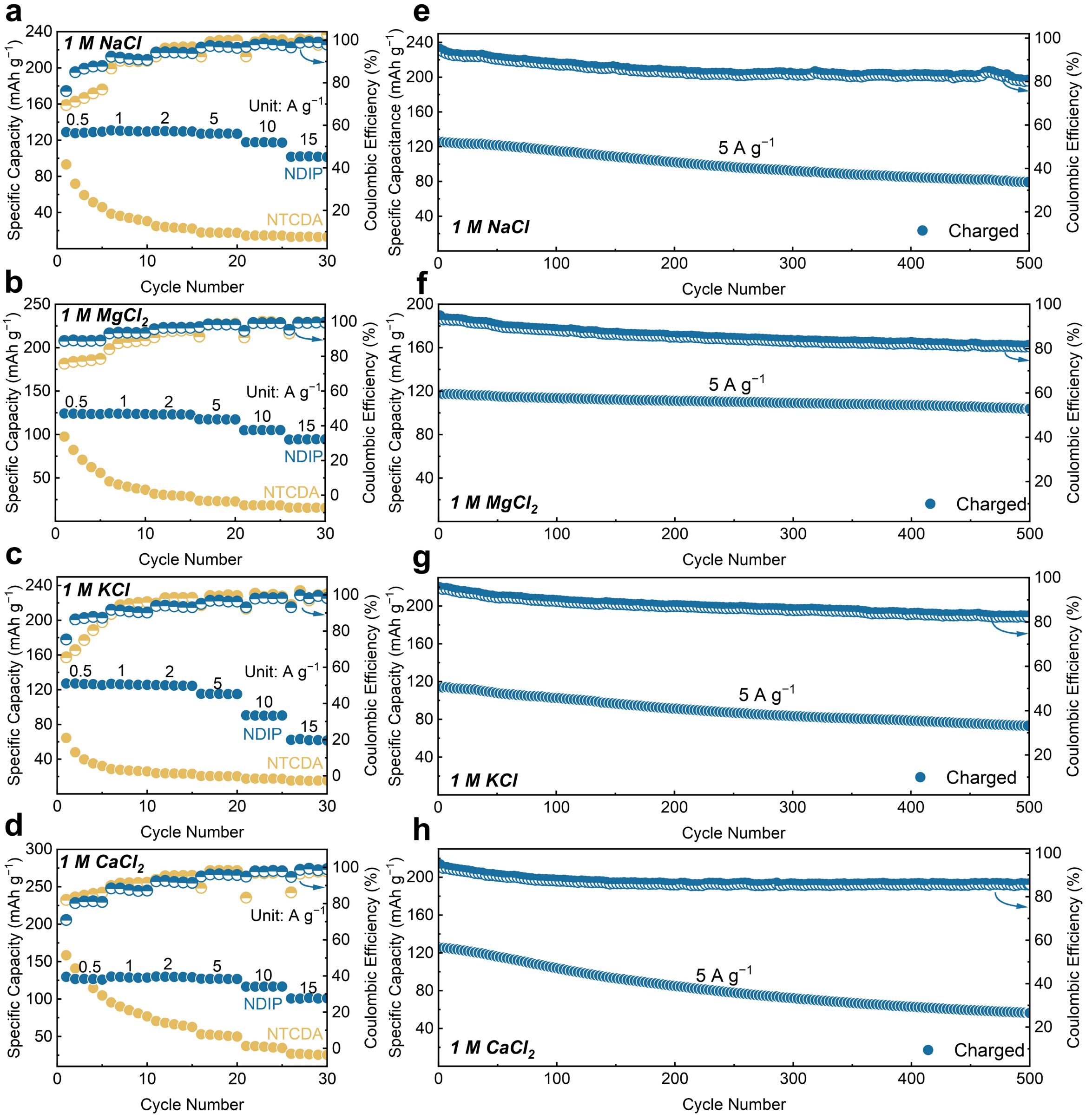

Figure S18. Rate performance of NDIP and NTCDA in (a) 1M NaCl, (b) 1 M $MgCl_2$, (c) 1 M KCl and (d) 1 M $CaCl_2$. Cyclic stability of NDIP in (e) 1M NaCl, (f) 1 M $MgCl_2$, (g) 1 M KCl and (h) 1 M $CaCl_2$.

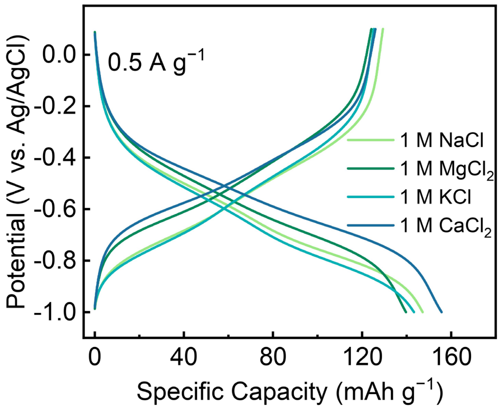

Figure S19. GCD profiles of NDIP at 0.5 A g^{-1} in different electrolytes.

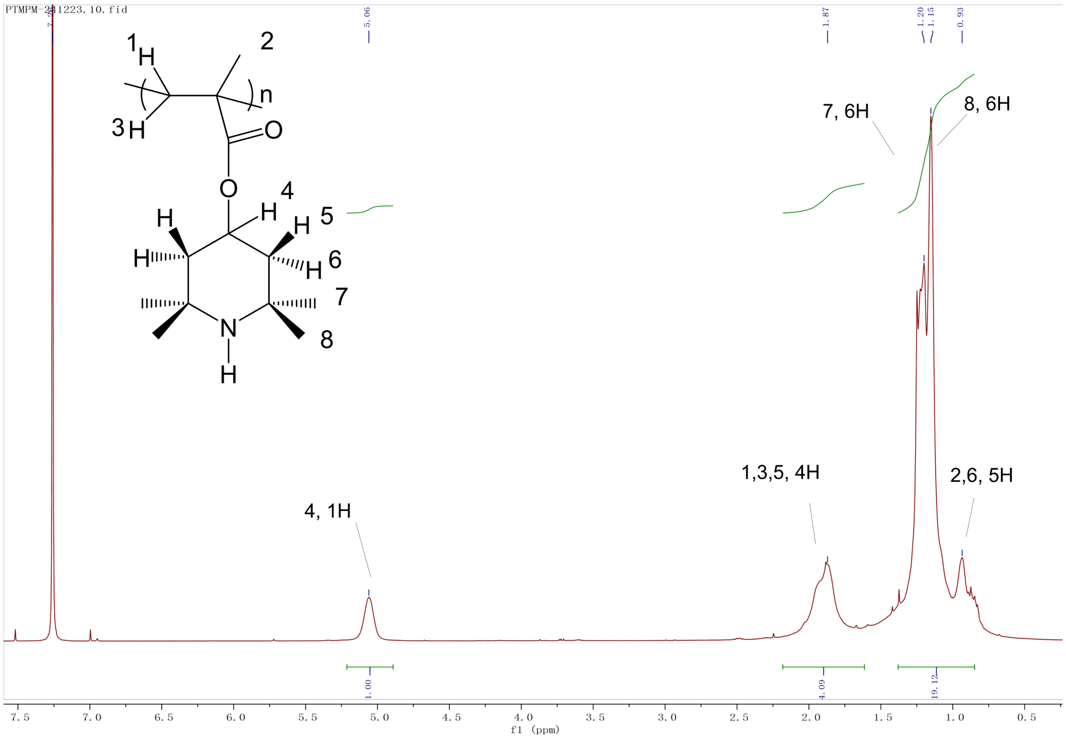

Figure S20. The ^{1}H NMR spectrum of PTMPM.

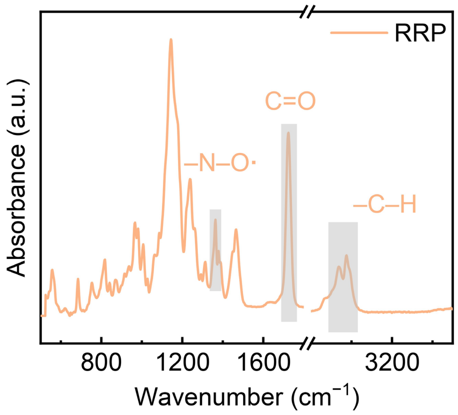

Figure S21. The ATR-FTIR spectrum of RRP.

Note: The peaks located at 2981/2947, 1726 and 1363 cm^{-1} are attributed to C–H, C=O, and N–O•, respectively, which confirm the successful synthesis of RRP.[S5]

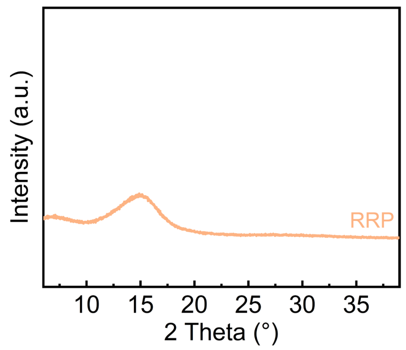

Figure S22. The PXRD pattern of RRP.

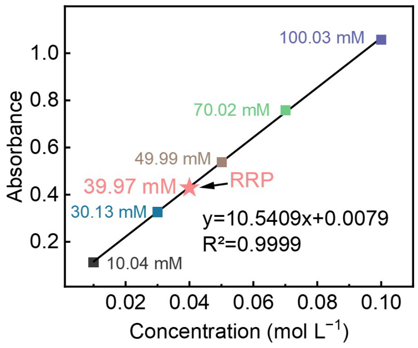

Figure S23. The fitted line of TEMPO-OH series solution and corresponding results of RRP based on peak intensity in UV spectrum.

Note: Owing to the exceptional solubility of RRP in dimethyl formamide, UV spectroscopy serves as an effective technique for quantifying the radical concentration of RRP relative to the reference TEMPO-OH solution. By implementing the peak intensity of RRP into the fitted line, the radical concentration in RRP is determined to be 89.80%.[S6]

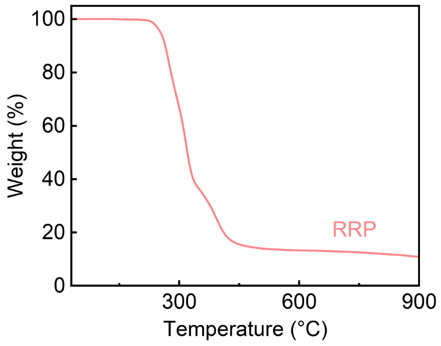

Figure S24. The TGA curve of RRP.

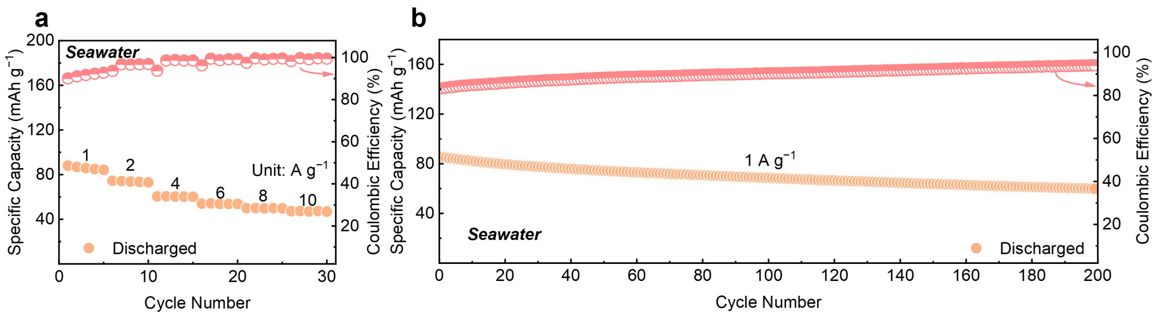

Figure S25. (a) The rate performance of RRP in seawater. (b) The cycle stability of RRP in seawater.

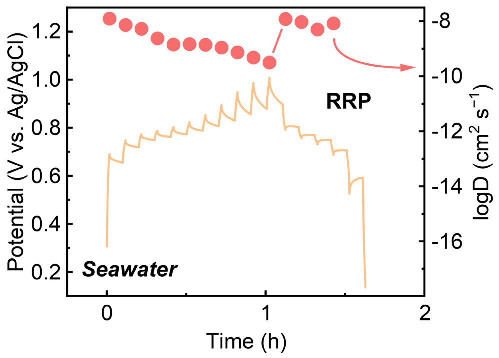

Figure S26. The GITT curves and the corresponding log(D) plots of RRP in seawater.

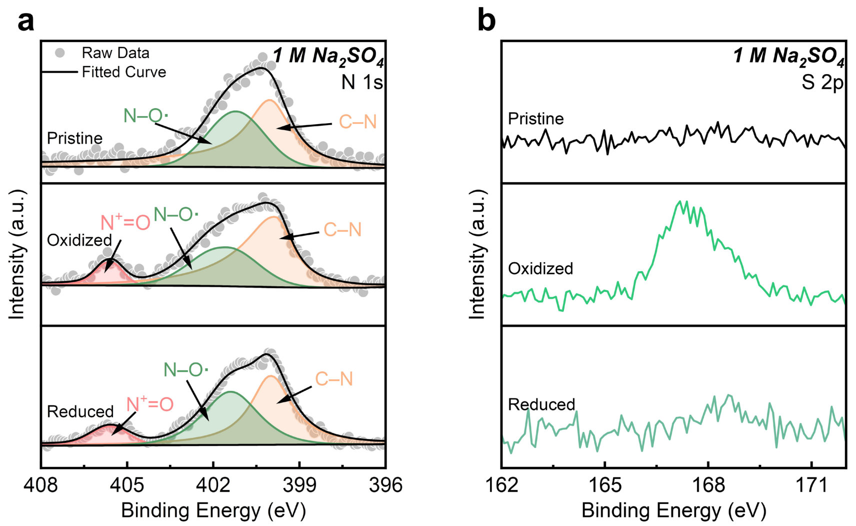

Figure S27. High-resolution of (a) N 1s and (b) S 2p XPS spectra of RRP-based electrode at pristine, oxidized and reduced states in 1 M Na_2SO_4.

Note: According to **Figure S27a**, the XPS N 1s spectra of RRP in 1 M Na_2SO_4 exhibit only two peaks at 400.1 and 401.1 eV, corresponding to C–N and N–O•, respectively. Following oxidation, a prominent peak at 405.6 eV indicates the emergence of N^+=O and the peak strength decreases subsequent to reduction. The ion coordination mechanism can be further validated by S 2p XPS profiles (**Figure S27b**), which indicates the interaction between N^+=O and the sulfate anions.[S7]

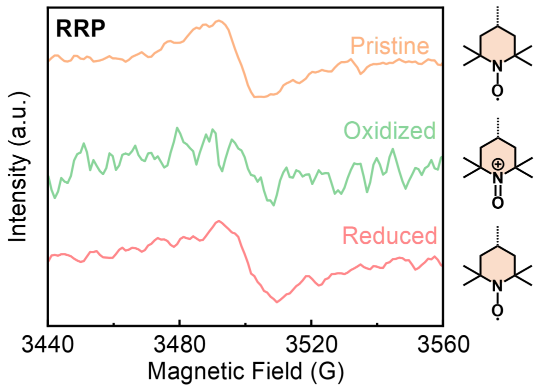

Figure S28. Ex-situ EPR spectra of RRP electrode at pristine, oxidized, and reduced states in seawater.

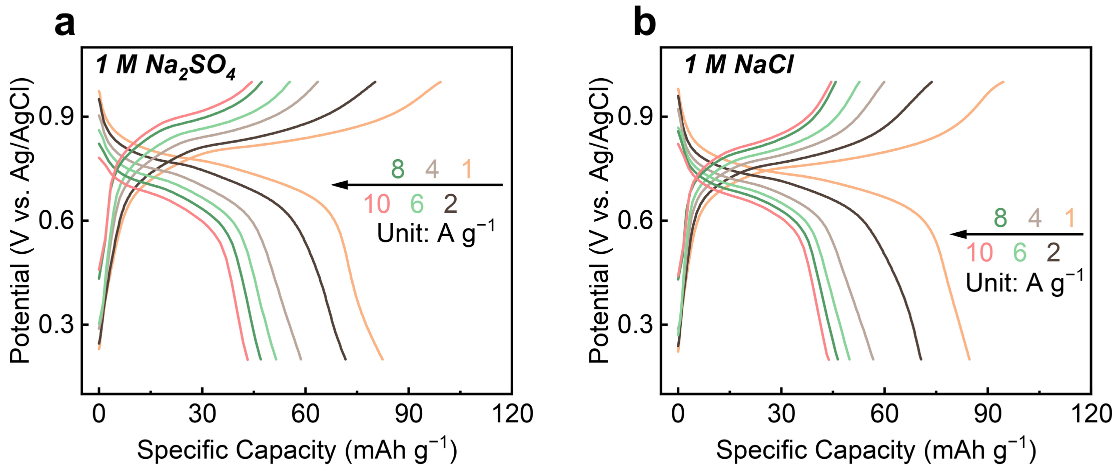

Figure S29. GCD curves of RRP at different current densities in (a) 1 M Na_2SO_4 and (b) 1 M NaCl.

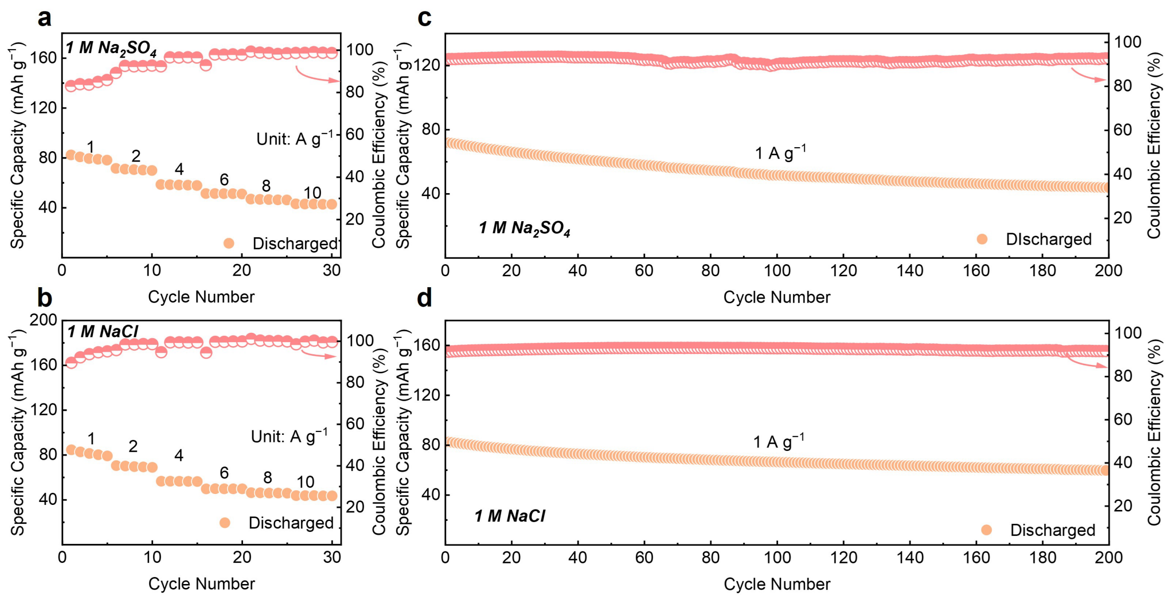

Figure S30. The rate performance of RRP in (a) 1 M Na_2SO_4 and (b) 1 M NaCl. The cycle stability of RRP in (c) 1 M Na_2SO_4 and (d) 1 M NaCl.

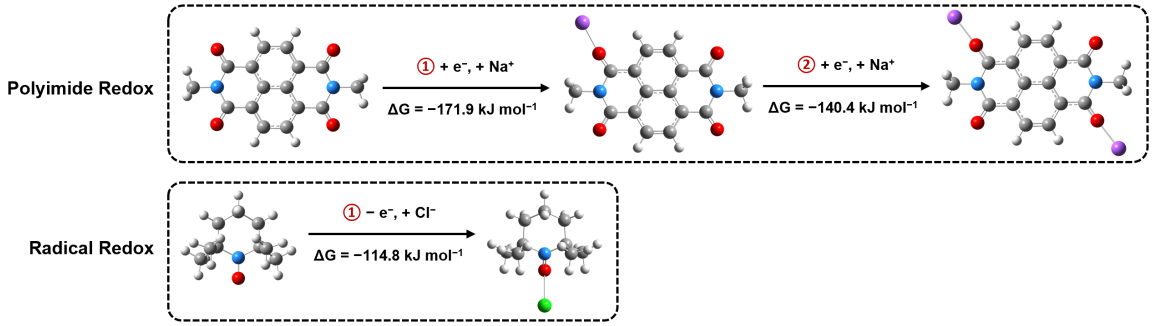

Figure S31. The possible mechanism and calculated free energy change for the redox reactions of NDIP and RRP fragments.

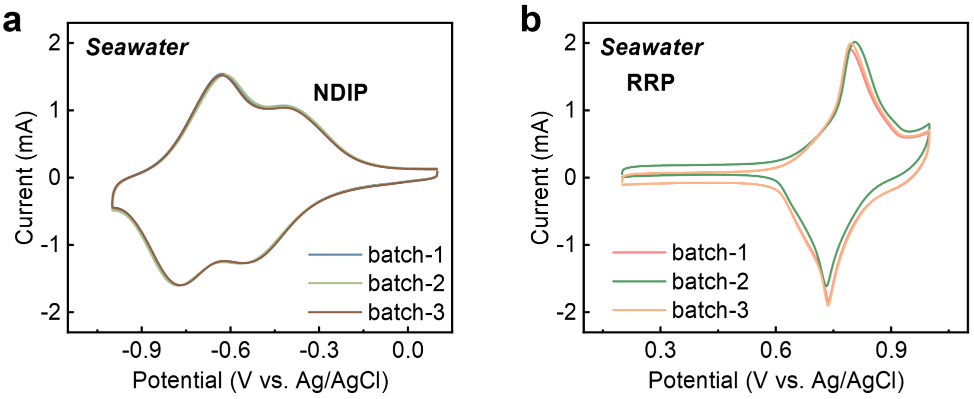

Figure S32. CV curves of (a) NDIP and (b) RRP in different batches of seawater.

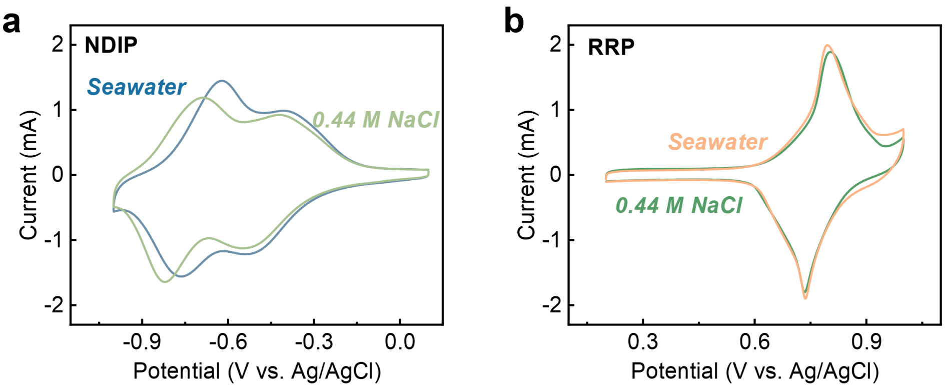

Figure S33. CV curves comparisons of (a) NDIP and (b) RRP in seawater and 0.44 M NaCl.

Note: 0.44 M NaCl solution possesses the similar amount of Na^+ and Cl^- with those in seawater.

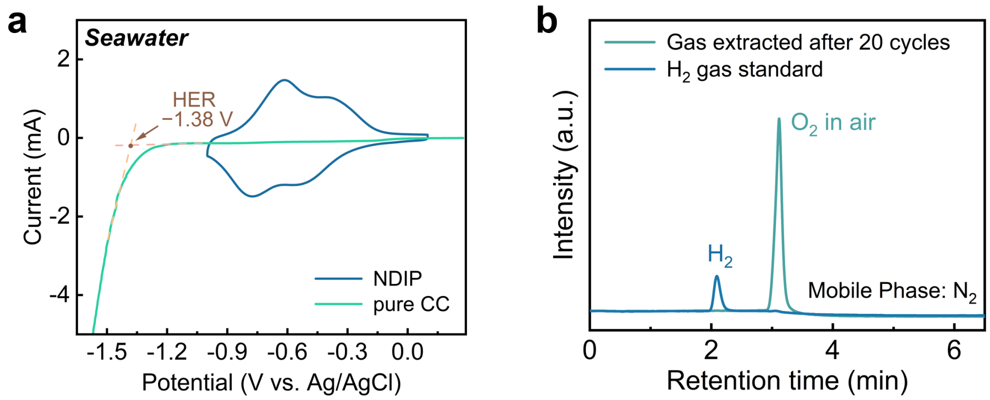

Figure S34. (a) CV curve of NDIP and LSV curve of pure CC in seawater (1 mV s^{-1}). (b) Gas chromatography-thermal conductivity detector spectra for the gas extracted after 20 redox cycles of NDIP and the pure hydrogen standard gas.

Note: Considering that our NDIP anode material is loaded on the carbon cloth (CC) current collector, we recorded its linear sweep voltammetry (LSV) curve to find out the HER onset potential (please see **Figure S34a**). It is observed that the HER onset potential of pure CC (−1.38 V vs. Ag/AgCl) is far more negative than the redox range of NDIP (−1.0–0.1 V vs. Ag/AgCl), implying the slight possibility for HER during the redox reaction of NDIP anode. We further tested NDIP at its redox range for 20 cycles in a sealed beaker cell and then extracted the inside gas for ex-situ gas chromatography (mobile phase is the N_2 gas). From the **Figure S34b** we can see that compared with the H_2 standard gas, only O_2 signal is observed (originated from air) and no H_2 can be detected, indicating the negligible HER at the anode side.

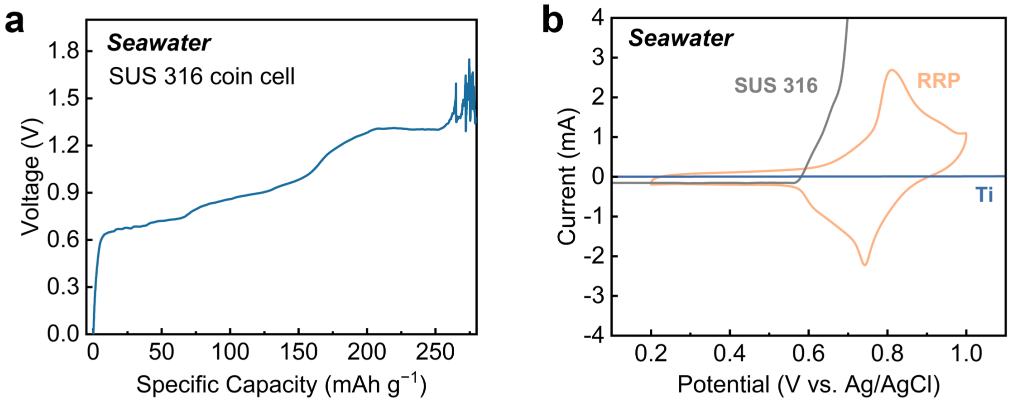

Figure S35. (a) The charged curve of NDIP//RRP coin cell (made of SUS 316). (b) The comparison of the CV curve of RRP and the LSV curves of SUS 316 and Ti.

Note: We assembled NDIP//RRP coin cells (made of SUS 316) and observe an abnormal charged curve at the high potential range, probably due to the corrosion of Cl^- on SUS material. We further conduct comparative LSV tests and found that SUS 316 shows a more negative corrosion onset potential than that of the redox peaks of RRP in seawater, while Ti (the current collector of the Swagelok cell is made of Ti) presents totally inactive behaviors.

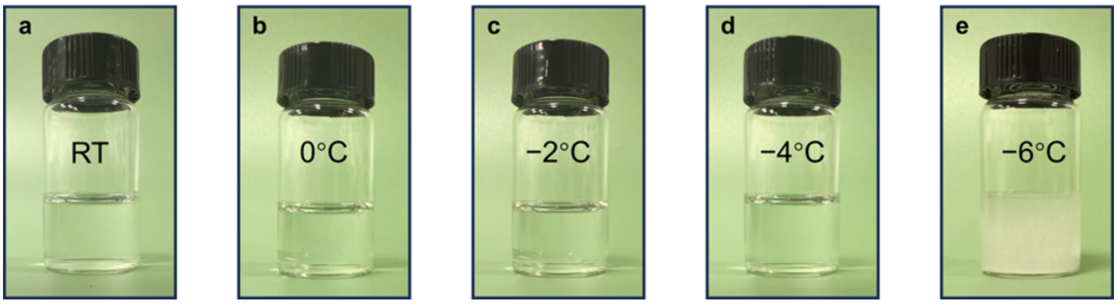

Figure S36. The digital images of seawater at (a) RT, (b) 0 °C, (c) −2 °C, (d) −4 °C and (e) −6 °C.

Note: While the commonly accepted freezing point of seawater is −2 °C, it is actually affected by salt concentration which differs by region (the higher the salt concentration in seawater, the lower the freezing point of seawater). The freezing point of our seawater (obtained from Lingshan Bay, Qingdao, China) is estimated to be around −6 °C. Thus, we designate −4 °C as the benchmark for low temperature tests.

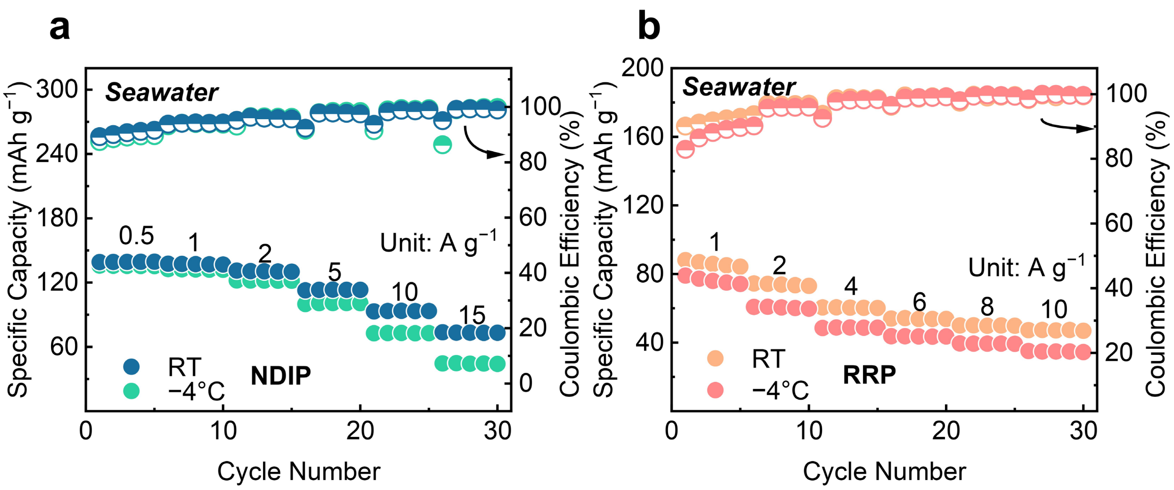

Figure S37. The rate performance of (a) NDIP and (b) RRP at room temperature and −4°C in seawater.

Note: The RT results of NDIP and RRP were adopted from **Figure 2E** and **Figure S25b**, respectively.

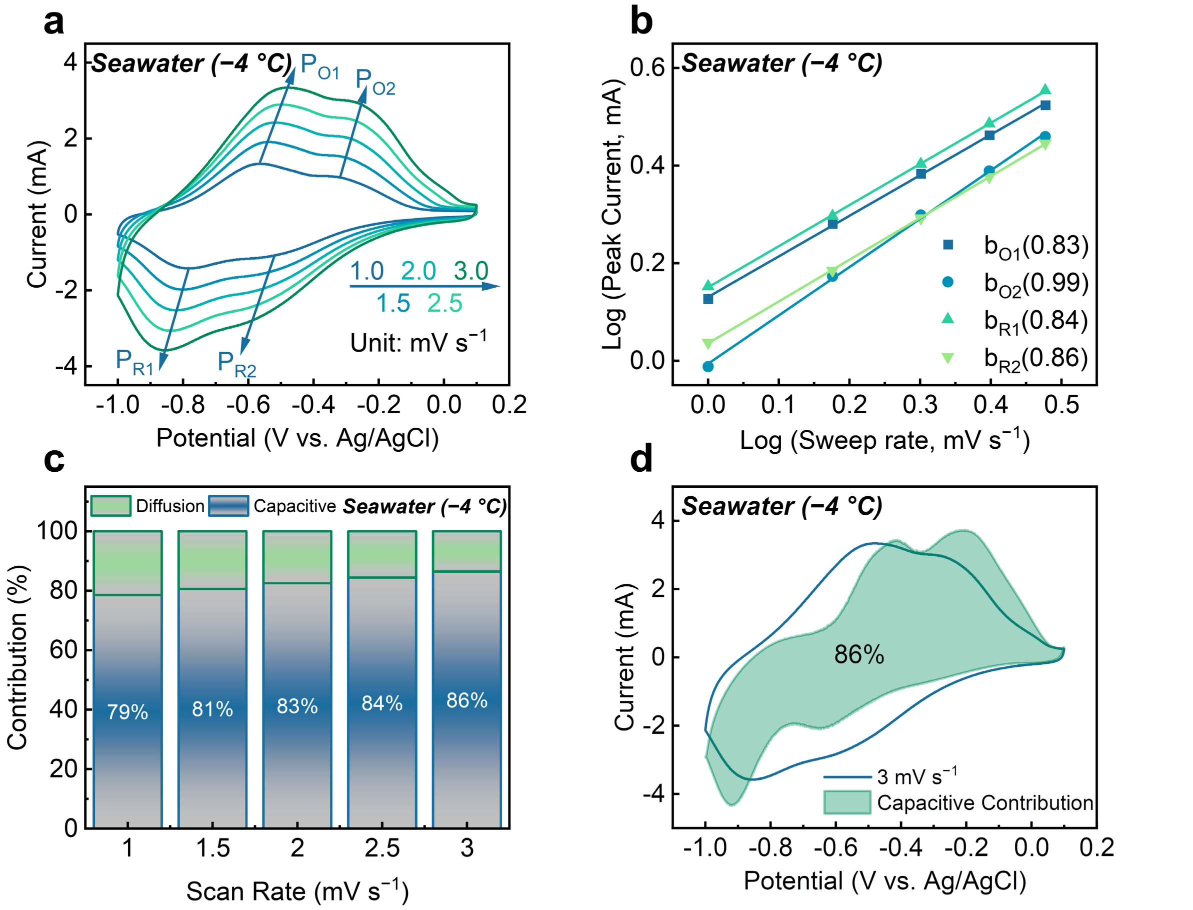

Figure S38. (a) CV curves at different scan rates; (b) b-value fitted lines; (c) column diagram of capacitance contribution and (d) the integral curve of capacitive contribution at 3.0 mV s^{-1} of NDIP in seawater at −4°C.

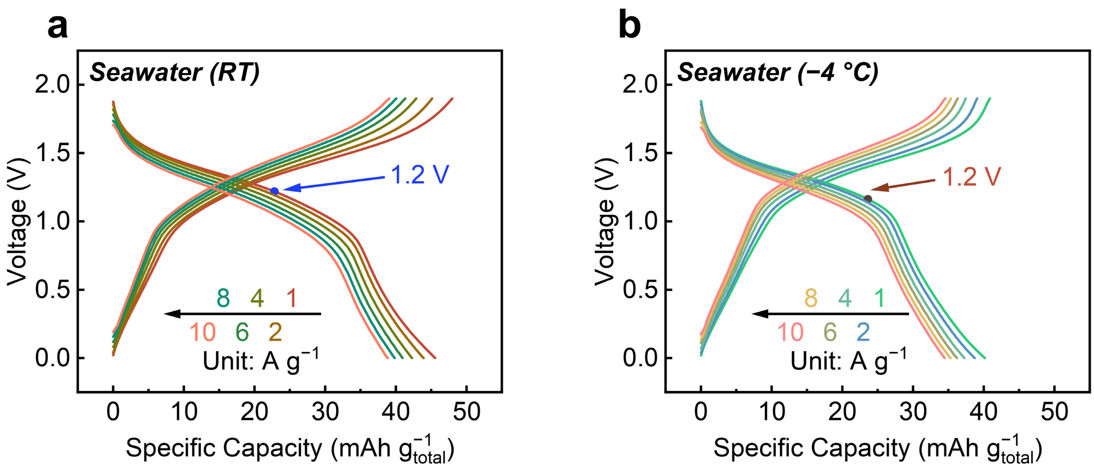

Figure S39. The GCD profiles of NDIP//RRP full battery at (a) room temperature and (b) −4°C.

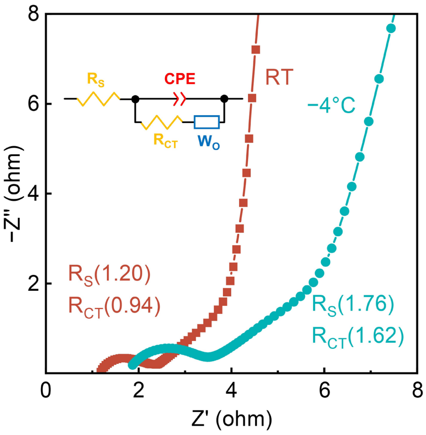

Figure S40. Nyquist plots and corresponding equivalent circuit diagram of NDIP//RRP batteries at RT and −4°C in seawater.

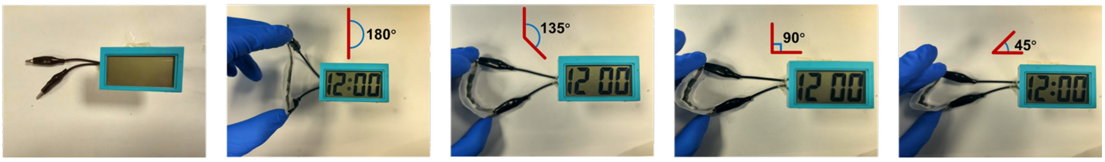

Figure S41. Pictures of the flexible NDIP//RRP battery lighting up a digital clock at various bending angles.

Table S1. Element analysis of NDIP

Sample	Element	Theoretical-1	Theoretical-2	Experimental
NDIP	C	65.50%	62.28%	62.17%
	N	12.07%	11.47%	11.21%
	H	4.06%	4.41%	3.97%
	O	18.37%	21.84%	21.44%

Note: The element content of theoretical-1 is calculated based on repeated unit (see details in Figure 2b). Considering that NDIP is composed of abundant hydrophilic N and O elements, which is prone to trap the moisture in air. Therefore, the element content of theoretical-2 is calculated based on the molecular formula $C_{19}H_{12}N_3O_3 \cdot H_2O$, which is more close to experimental result.[S8]

Table S2. Molecular weight, polydispersity, and degree of polymerization of RRP

Sample	Mn	Mw	Polydispersity	Degree of Polymerization
RRP	175969	553794	3.15	732

Table S3. Element analysis of RRP

Sample	Element	Theoretical	Experimental
RRP	C	65.00%	62.60%
	N	5.83%	5.53%
	H	9.04%	9.17%
	O	20.00%%	20.76%

Table S4. Summary of capacity retention of the electrodes for seawater applications

Electrode	Electrolyte	Retention@Cycles	Reference No.
PGNPC	Seawater	93.7%@5000	[S9]
h-PPy	Seawater	99.3%@6000	[S10]
Zn-Mn alloy	2 M ZnSO4 in seawater	99.6%@2500	[S11]
FeHCF	Seawater	~100%@50	[S12]
NiHCF	Seawater	61.4%@200	[S13]
$NaTi_2(PO_4)_3/C$	Seawater	65.6%@1000	[S14]
$K_{0.97}Co_{0.8}Mn_{0.2}[Fe(CN)_6]_{0.81}\cdot 2.2H_2O$	Seawater	87.6%@1000	[S14]
E-TiO_2	1 M NaCl	~85%@3000	[S15]
NDIP	1 M Na_2SO_4	95.5%@10000	This work
NDIP	Seawater	84.2%@10000	This work

Table S5. Summary of the specific capacity of the electrodes in seawater or aqueous Na^+/Mg^{2+}/K^+/Ca^{2+} electrolytes

**Electrode**	**Na^+**	**K^+**	**Mg^{2+}**	**Ca^{2+}**	**Seawater**	**Reference No.**
E-TiO_2	142.0	13.5	36.6	49.7	115	[S15]
K-FeHCF	110.0	121.0	n.a.	n.a.	n.a.	[S16]
Ni_1Zn_1HCF	59.7	61.9	n.a.	n.a.	n.a.	[S17]
CuHCF	60.4	60.5	n.a.	n.a.	n.a.	[S18]
InHCF	53.9	53.3	n.a.	n.a.	n.a.	[S19]
NTCDA	42.1	32.2	55.8	104.8	37.4	This work
NDIP	148.9	125.3	123.4	126.0	138.1	This work

Note: The unit is mAh g^{-1}. The specific capacities of E-TiO_2 in Na^+ and seawater is based the corresponding descriptions in the literature, while its specific capacity of Mg^{2+}/K^+/Ca^{2+} is converted from areal capacities (μAh cm^{-2}) to gravimetric specific capacities (mAh g^{-1}) according to corresponding descriptions and figures in the literature.

Table S6. Major ion composition and pH of different batches of seawater

Ingredient	Batch 1 (mg L^{-1})	Batch 2 (mg L^{-1})	Batch 3 (mg L^{-1})
Chloride (Cl^-)	17100	17900	17700
Sodium (Na^+)	10100	10500	10400
Sulfate (SO_4^{2-})	2530	2590	2540
Magnesium (Mg^{2+})	1200	1250	1220
Potassium (K^+)	376	393	379
Calcium (Ca^{2+})	370	380	370
Bromide (Br^-)	48	50	55
pH	7.60	7.56	7.59

Table S7. Comparison of the average output voltage with other aqueous polymer-air batteries

Configuration	Electrolyte	Average Output Voltage	Reference No.
BBL@CNT//Air	0.5 M H_2SO_4	0.47 V	[S20]
pEP(NQ)E//Air	0.5 M H_2SO_4	0.50 V	[S21]
PDBM@SWNT//Air	0.5 M H_2SO_4	0.55 V	[S22]
PVAQ//Air	30 wt % KOH	0.56 V	[S23]
AQ modified CO-POSS//Air	0.1 M NaOH	0.66 V	[S24]
NHCC//Air	4 M KOH+10 M KCF_3SO_3	0.70 V	[S25]
P14AQ@CNT//Air	6 M KOH	0.73 V	[S26]
PNQ-carbon composite//Air	0.5 M H_2SO_4	0.73 V	[S27]
PQNB//Air	10 M NaOH	0.73 V	[S28]
NDIP//RRP (−4°C)	Seawater	1.20 V	This work

Table S8. Comparison of the capacity retention and energy efficiency between this work and other seawater batteries based on air cathodes

Anode	Anolyte	Cathode	Catholyte	Retention @Cycles	Energy Efficiency	Reference No.
E-TiO_2	seawater	Air Catalyst	seawater	52.2%@600	25.5%	[S15]
P/C@Na-BP-PYRs	1 M $NaPF_6$ in DEGDME	Air Catalyst	seawater	99.0%@50	52.2%	[S29]
PC composite	1 M $NaClO_4$ in EC/DEC	Air Catalyst	seawater	93.2%@80	40.6%	[S30]
Hard Carbon	1 M $NaCF_3SO_3$ in TEGDME	Air Catalyst	seawater	82.4%@100	51.4%	[S31]
HC-PSS	1 M $NaCF_3SO_3$ in TEGDME	Air Catalyst	seawater	99.7%@100	49.2%	[S32]
Sn-C	ILE	Air Catalyst	seawater	92.0%@50	53.2%	[S33]
NDIP	seawater@−4°C	RRP	Seawater @−4°C	83.2%@800	81.0%	This work

References

[S1] F. Huang, W. Zhao, Y. Guo, Y. Mi, S. Gull, G. Long, P. Du, *Adv. Funct. Mater.* **2024**, 34, 2407313.

[S2] S. Cui, T. Li, D. Tao, D. Zhang, Y. Cao, F. Xu, *Energy Storage Mater.* **2024**, 71, 103672.

[S3] J. Qin, Y. Liu, G. Li, Q. Lan, Z. Song, X. Ai, H. Zhan, *Adv. Energy Mater.* **2024**, 14, 2400731.

[S4] D. W. Dees, S. Kawauchi, D. P. Abraham, J. Prakash, *J. Power Sources* **2009**, 189, 263.

[S5] W. Guo, Y.-X. Yin, S. Xin, Y.-G. Guo, L.-J. Wan, *Energy Environ. Sci.* **2012**, 5, 5221.

[S6] K. Zhang, Y. Hu, L. Wang, J. Fan, M. J. Monteiro, Z. Jia, *Polym. Chem.* **2017**, 8, 1815.

[S7] T. Zhou, W. Jin, W. Xue, B. Dai, C. Feng, X. Huang, P. Théato, Y. Li, *J. Power Sources* **2021**, 483, 229136.

[S8] Z. Meng, A. Aykanat, K. A. Mirica, *Chem. Mater.* **2019**, 31, 819.

[S9] P. Zhao, M. Yao, Q. Zhang, N. Wang, W. Hu, S. Komarneni, *Electrochim. Acta* **2019**, 318, 211.

[S10] B. Zhang, C. Zhang, W. Yuan, O. Yang, Y. Liu, L. He, Y. Hu, L. Zhou, J. Wang, Z. L. Wang, *ACS Appl. Mater. Interfaces* **2022**, 14, 9046.

[S11] H. Tian, Z. Li, G. Feng, Z. Yang, D. Fox, M. Wang, H. Zhou, L. Zhai, A. Kushima, Y. Du, Z. Feng, X. Shan, Y. Yang, *Nat. Commun.* **2021**, 12, 237.

[S12] Y. Liu, L. Luo, Z. Shen, Y. Ji, Z. Wen, Z. Li, J. Li, P. Sun, J. Xie, G. Hong, *Chem. Eng. J.* **2023**, 465, 142733.

[S13] S. T. Senthilkumar, M. Abirami, J. Kim, W. Go, S. M. Hwang, Y. Kim, *J. Power Sources* **2017**, 341, 404.

[S14] J. Wu, Y. Zheng, P. Zhang, X. Rao, Z. Zhang, J.-M. Wu, W. Wen, *Research* **2024**, 7, 0461.

[S15] W. Wen, C. Geng, X. Li, H. Li, J.-M. Wu, H. Kobayashi, T. Sun, Z. Zhang, D. Chao, *Adv. Mater.* **2024**, 36, 2312343.

[S16] C. Liu, X. Wang, W. Deng, C. Li, J. Chen, M. Xue, R. Li, F. Pan, *Angew. Chem. Int. Ed.* **2018**, 57, 7046.

[S17] L. Chen, L. Zhang, X. Zhou, Z. Liu, *ChemSusChem* **2014**, 7, 2295.

[S18] P. Jiang, H. Shao, L. Chen, J. Feng, Z. Liu, *J. Mater. Chem. A* **2017**, 5, 16740.

[S19] L. Chen, H. Shao, X. Zhou, G. Liu, J. Jiang, Z. Liu, *Nat. Commun.* **2016**, 7, 11982.

[S20] T. Ma, Y. Yang, D. Johnson, K. Hansen, S. Xiang, R. M. Thakur, A. Djire, J. L. Lutkenhaus, *Joule* **2023**, 7, 2261.

[S21] K. Oka, C. Strietzel, R. Emanuelsson, H. Nishide, K. Oyaizu, M. Strømme, M. Sjödin, *ChemSusChem* **2020**, 13, 2280.

[S22] K. Oka, S. Furukawa, S. Murao, T. Oka, H. Nishide, K. Oyaizu, *Chem. Commun.* **2020**, 56, 4055.

[S23] W. Choi, D. Harada, K. Oyaizu, H. Nishide, *J. Am. Chem. Soc.* **2011**, 133, 19839.

[S24] R. Tanaka, S. Kitajima, N. Tohnai, K. Oka, H. Imoto, K. Naka, *ChemNanoMat* **2024**, 10, e202400122.

[S25] S. Li, S. Hu, H. Li, C. Han, *Angew. Chem. Int. Ed.* **2024**, 63, e202318885.

[S26] Y. Li, L. Liu, C. Liu, Y. Lu, R. Shi, F. Li, J. Chen, *Chem* **2019**, 5, 2159.

[S27] K. Oka, S. Murao, K. Kobayashi, H. Nishide, K. Oyaizu, *ACS Appl. Energy Mater.* **2020**, 3, 12019.

[S28] T. Kawai, K. Oyaizu, H. Nishide, *Macromolecules* **2015**, 48, 2429.

[S29] Y. Kim, A. Varzi, A. Mariani, G.-T. Kim, Y. Kim, S. Passerini, *Adv. Energy Mater.* **2021**, 11, 2102061.

[S30] Y. Kim, S. M. Hwang, H. Yu, Y. Kim, *J. Mater. Chem. A* **2018**, 6, 3046.

[S31] H. Kim, J.-S. Park, S. H. Sahgong, S. Park, J.-K. Kim, Y. Kim, *J. Mater. Chem. A* **2014**, 2, 19584.

[S32] D. H. Lim, C. Dong, H. W. Kim, G. H. Bae, K. Choo, G. B. Cho, Y. Kim, B. Jin, J. K. Kim, *Mater. Today Energy* **2021**, 21, 100805.

[S33] J.-K. Kim, F. Mueller, H. Kim, S. Jeong, J.-S. Park, S. Passerini, Y. Kim, *ChemSusChem* **2016**, 9, 42.
